# Physiological and transcriptome response to cadmium in cosmos (*Cosmos bipinnatus* Cav.) seedlings

**DOI:** 10.1038/s41598-017-14407-8

**Published:** 2017-10-31

**Authors:** Yujing Liu, Xiaofang Yu, Yimei Feng, Chao Zhang, Chao Wang, Jian Zeng, Zhuo Huang, Houyang Kang, Xing Fan, Lina Sha, Haiqin Zhang, Yonghong Zhou, Suping Gao, Qibing Chen

**Affiliations:** 1 Landscape Architecture, Sichuan Agricultural University, Wenjiang, 611130 Sichuan China; 2 Industrial Crop Research Institute of Sichuan Academy of Agricultural Sciences, Qingbaijiang, 610300 Sichuan China; 3 Triticeae Research Institute, Sichuan Agricultural University, Wenjiang, 611130 Sichuan China; 4 College of Resources, Sichuan Agricultural University, Wenjiang, 611130 Sichuan China

## Abstract

To date, several species of *Asteraceae* have been considered as Cd-accumulators. However, little information on the Cd tolerance and associated mechanisms of *Asteraceae* species *Cosmos bipinnatus*, is known. Presently, several physiological indexes and transcriptome profiling under Cd stress were investigated. *C. bipinnatus* exhibited strong Cd tolerance and recommended as a Cd-accumulator, although the biomasses were reduced by Cd. Meanwhile, Cd stresses reduced Zn and Ca uptake, but increased Fe uptake. Subcellular distribution indicated that the vacuole sequestration in root mainly detoxified Cd under lower Cd stress. Whilst, cell wall binding and vacuole sequestration in root co-detoxified Cd under high Cd exposure. Meanwhile, 66,407 unigenes were assembled and 41,674 (62.75%) unigenes were annotated in at least one database. 2,658 DEGs including 1,292 up-regulated unigenes and 1,366 down-regulated unigenes were identified under 40 μmol/L Cd stress. Among of these DEGs, *ZIPs*, *HMAs*, *NRAMPs* and *ABC* transporters might participate in Cd uptake, translocation and accumulation. Many DEGs participating in several processes such as cell wall biosynthesis, GSH metabolism, TCA cycle and antioxidant system probably play critical roles in cell wall binding, vacuole sequestration and detoxification. These results provided a novel insight into the physiological and transcriptome response to Cd in *C. bipinnatus* seedlings.

## Introduction

Cadmium (Cd), a non-essential heavy metal, causes a distinct toxicity in both plants and humans^1^. In *planta*, Cd directly or indirectly causes several toxicities, such as inducing oxidative stress^2–4^, altering the chloroplast ultrastructure^5^, damaging chlorophyll synthesis, impairing photosynthetic efficiency^6,7^, and reducing mineral nutrient uptake such as Zn, Fe, and Ca^8^, finally inhibiting plant growth and causing death^9–11^. However, some Cd- tolerance plants or hyper-accumulators such as *Thlaspi caerulescens* ^12^, *Sedum alfredii* ^13^, *Viola baoshanensis* ^14^, and *Solanum nigrum* ^15^ accumulate high Cd concentrations in shoots without or having only mild toxicity symptoms^16^, which therefore have been/being used for phytoremediation of Cd. Meanwhile, their physiological and molecular mechanisms of Cd tolerance have been/being substantially revealed^17–19^. However, different species exhibit different Cd uptake, translocation, detoxification and their associated mechanisms. Thus, it is crucial to identify new Cd accumulators or hyper-accumulators, and understand their physiological and molecular mechanism.

Several species of the *Asteraceae* family, such as *Crassocephalum crepidioides* ^20^, *Bidens pilosa*, *Kalimeris integrifolia* ^21^, *Chromolaena odorata* ^22^, *Elephantopus mollis* ^23^, and *Picris divaricata* ^24^, are recommended as Cd-accumulators, which are used for phytoremediation. Cosmos (*Cosmos bipinnatus* Cav.), an annual species of *Asteraceae*, possesses ornamental value in its leaves and flowers, as well as strong adaption and plasticity traits in adverse environments. Thus, it is now widely cultivated in China. Previous study indicated that *C. bipinnatus* is a potential chromium (Cr) hyper-accumulator in plants^25^. Whether is it a Cd hyperaccumulator/accumulator, and possesses unique physiological and molecular mechanisms?

With the advent of next-generation sequencing (NGS) technology, RNA sequencing (RNA-Seq) has been/being widely used to reveal molecular mechanisms under abiotic stresses and to enrich our transcriptional evidence for plants^26,27^. Increasing studies using RNA-Seq have revealed Cd response in different plants and understood their associated molecular mechanism^28–31^. For example, compared with low-Cd-accumulation (LCA) genotypes, transcriptomic evidence indicated that high-Cd-accumulation (HCA) genotypes have more complicated mechanisms when exposed to Cd^32–34^. Additionally, RNA-Seq has also been used to screen candidate genes for Cd hyper-accumulator and provide a novel perspective on the molecular mechanisms, such as in *Noccaea caerulescens* ^35^ and *Solanum nigrum* ^36^. However, the transcriptome information for *C. bipinnatus* under Cd stress, is still unknown.

In the present study, we identified a new Cd accumulator, *C. bipinnatus*, from the *Asteraceae* family, and aimed to reveal its potential physiological and molecular mechanisms using metal subcellular distribution, several biochemical indexes, and RNA-Seq. Additionally, due to lack of genomic information of *C. bipinnatus*, construction of the transcriptome of the *C. bipinnatus* would facilitate its molecular research.

## Results

### Plant growth

**Figure 1:**
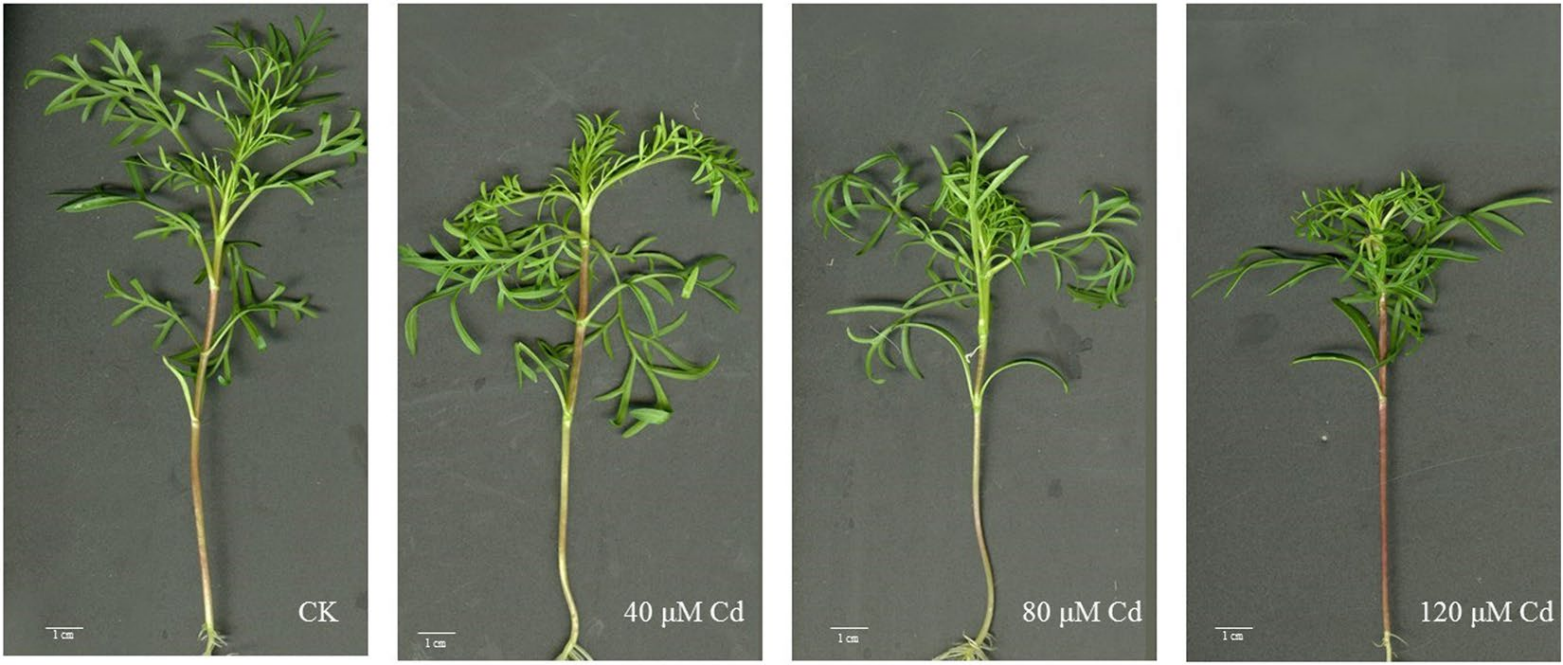
Growth of *C. bipinnatus * treated with different Cd concentrations.

**Figure 2:**
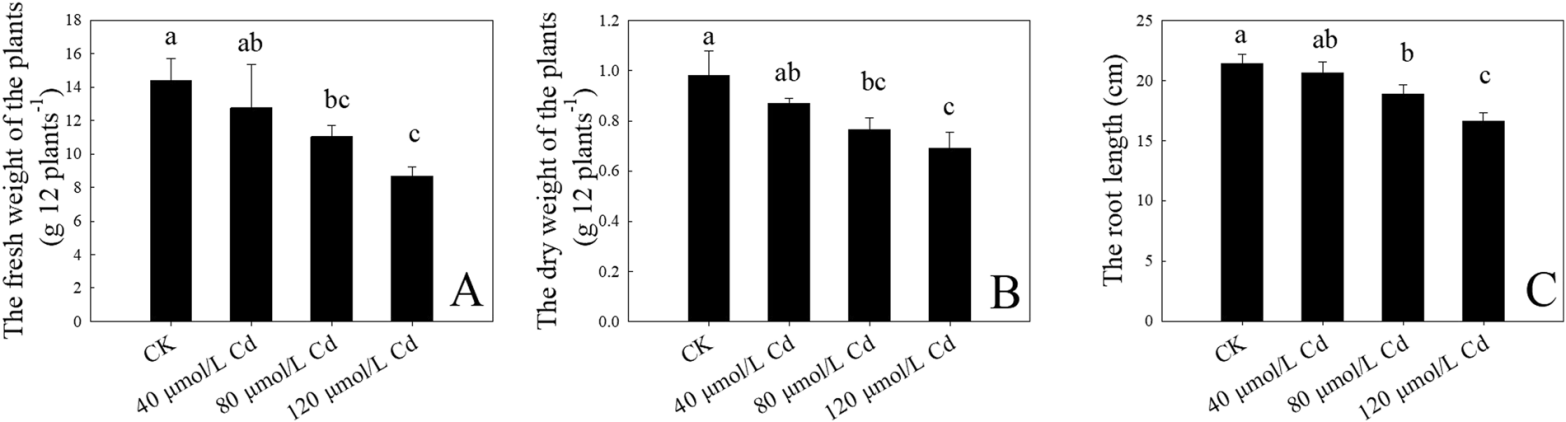
The growth of *C. bipinnatus* exposed to Cd. A: the fresh weight of the plants; B: the dry weight of the plants, and C: the root length. Values were means ± standard deviation (n = 3); values followed by different lowercase letters show significant differences at *P* < 0.05 .

Compared with control, the seedlings treated with 40 μmol/L Cd did not show obvious toxicity symptoms after 9 days of treatment, while 80 and 120 μmol/L Cd treatment exhibited visibly toxic symptoms, such as the decreased leaf number, and the reddened stems at 120 μmol/L (Fig. 1). 40 μmol/L Cd did not significantly affect the fresh and dry weight of plant and the length of root (Fig. 2A–C). However, the biomass was significantly reduced by 80 and 120 μmol/L Cd (Fig. 2A and B). Since several significant changes were observed between 40 and 80 μmol/L (Fig. 1 and 2), 40 μmol/L Cd should be recommended as the threshold of normal growth.

### Cd accumulation and distribution

The Cd concentration was not observed in all samples under 0 μmol/L Cd stress (Table 1). The Cd concentrations of all samples increased significantly with increasing Cd concentration. Cd accumulated highly in the roots, followed by the stems and the leaves (Table 1). Translocation factor (TF) values of the stems ranged from 0.56–0.64 was higher than those of the leaves ranged from 0.19–0.29 (Table 1), suggesting that most of Cd in aboveground was sequestrated in the stems.

**Table 1:** The concentration of Cd in dry tissues and translocation factor (TF) of *C. bibinnatus* seedlings treated with different levels of Cd.

Treatment	Leaf (μg/g DW)	Stem (μg/g DW)	Root (μg/g DW)	TF	
				Stem	Leaf
CK	N.D.	N.D.	N.D.	N.D.	N.D.
40 μmol/L Cd	60.36 ± 2.17a	321.15 ± 16.04b	576.65 ± 41.48b	0.56	0.19
80 μmol/L Cd	93.41 ± 8.29b	414.23 ± 25.64ab	648.98 ± 55.83b	0.64	0.23
120 μmol/L Cd	145.87 ± 6.73c	499.05 ± 87.54a	806.07 ± 36.26a	0.62	0.29

Values are mean ± standard deviation (n = 3). Values within a column followed by different lowercase letters show significant differences at *P* < 0.05. N.D., not detected under the detection limit of Cd: 2.5 μg/g, the same as below. TF = [the mean value of concentration in stems]/[the mean value of concentration in roots] for stems and [the mean value of concentration in leaves]/[the mean value of concentration in roots] for leaves.

**Figure 3:**
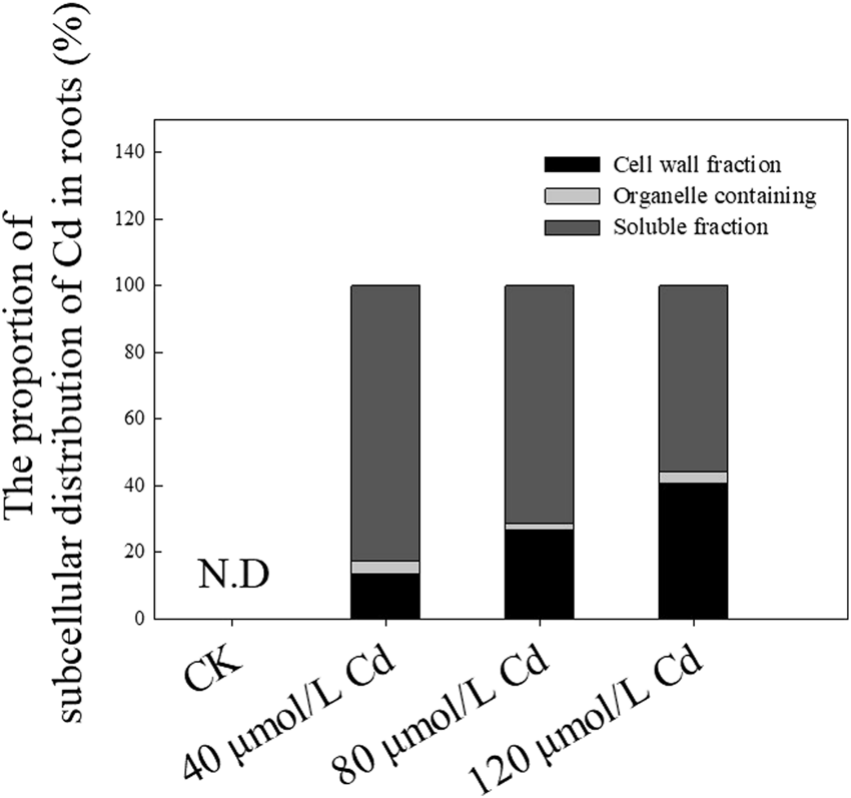
The subcellular distribution of Cd under different concentrations of Cd.

In order to understand whether different Cd stresses exhibited different Cd detoxifications or toxicity, we analyzed the Cd subcellular distribution mainly in the roots under these three Cd stresses. Under 40 μmol/L Cd stress, more than 80% Cd was accumulated in the soluble fraction, approximate 15% Cd was accumulated in the cell wall fraction, and only 5% Cd was transported into the organelle fraction (Fig. 3). Although Cd in soluble fraction was dramatically reduced with the increasing Cd concentrations, more than 55% Cd was still sequestrated in this fraction when treated with 120 μmol/L Cd (Fig. 3). Meanwhile, Cd in cell wall fractions were significantly increased with the increasing Cd concentrations, up to 40% Cd was binding in cell wall fraction when treated with 120 μmol/L Cd. These results indicated that the sequestration of Cd into soluble fraction is the main Cd detoxification mechanism under low Cd stress, while the sequestration of Cd into soluble fraction and the binding of Cd in the cell wall fraction represent a coaction for Cd detoxification with the increasing Cd concentrations.

### Effects of Cd on Zn, Ca, and Fe concentrations in *C. bipinnatus*

**Figure 4:**
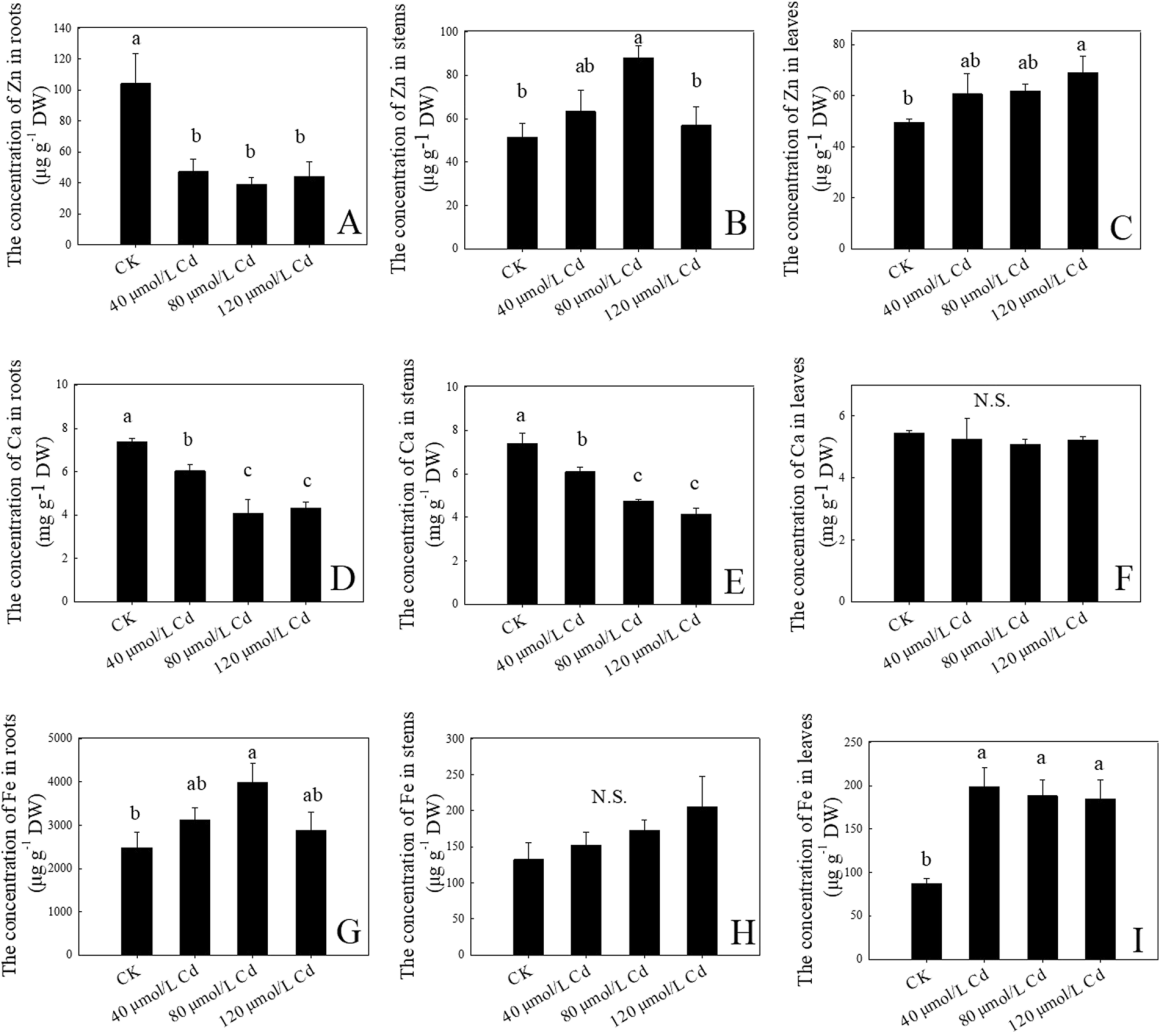
The concentration of metals under different Cd treatments of *C. bipinnatus* seedlings. A, B, and C: the concentration of Zn; D, E, and F: the concentration of Ca; G, H, and I: the concentration of Fe. N.S., no difference in various treatments. Values were means ± standard deviation (n = 3); values followed by different lowercase letters show significant differences at *P* < 0.05.

After 9 days of treatments, the Cd stresses significantly decreased the uptake of Zn in the roots when compared with CK (Fig. 4A). Interestingly, Zn concentration in the stems and leaves were mainly increased, although some decreased at 120 μmol/L Cd in stems (Fig. 4B and C). These results indicated that Cd inhibited the uptake of Zn in the roots, but promoted the translocation of Zn from root to shoot. Meanwhile, Cd stresses significantly decreased the Ca concentration in the stems and the roots (Fig. 4D and E), but did not affect the Ca concentration in the leaves (Fig. 4F). Cd increased Fe concentrations in roots and stems leaves, although the differences were not significant (Fig. 4G and H). But it significantly increased the Fe concentrations in leaves (Fig. 4I). These results indicated that Cd treatment may differentially affect the uptake of metal nutrients in *C. bipinnatus*.

### MDA concentrations and the activity of several antioxidant enzymes

**Figure 5:**
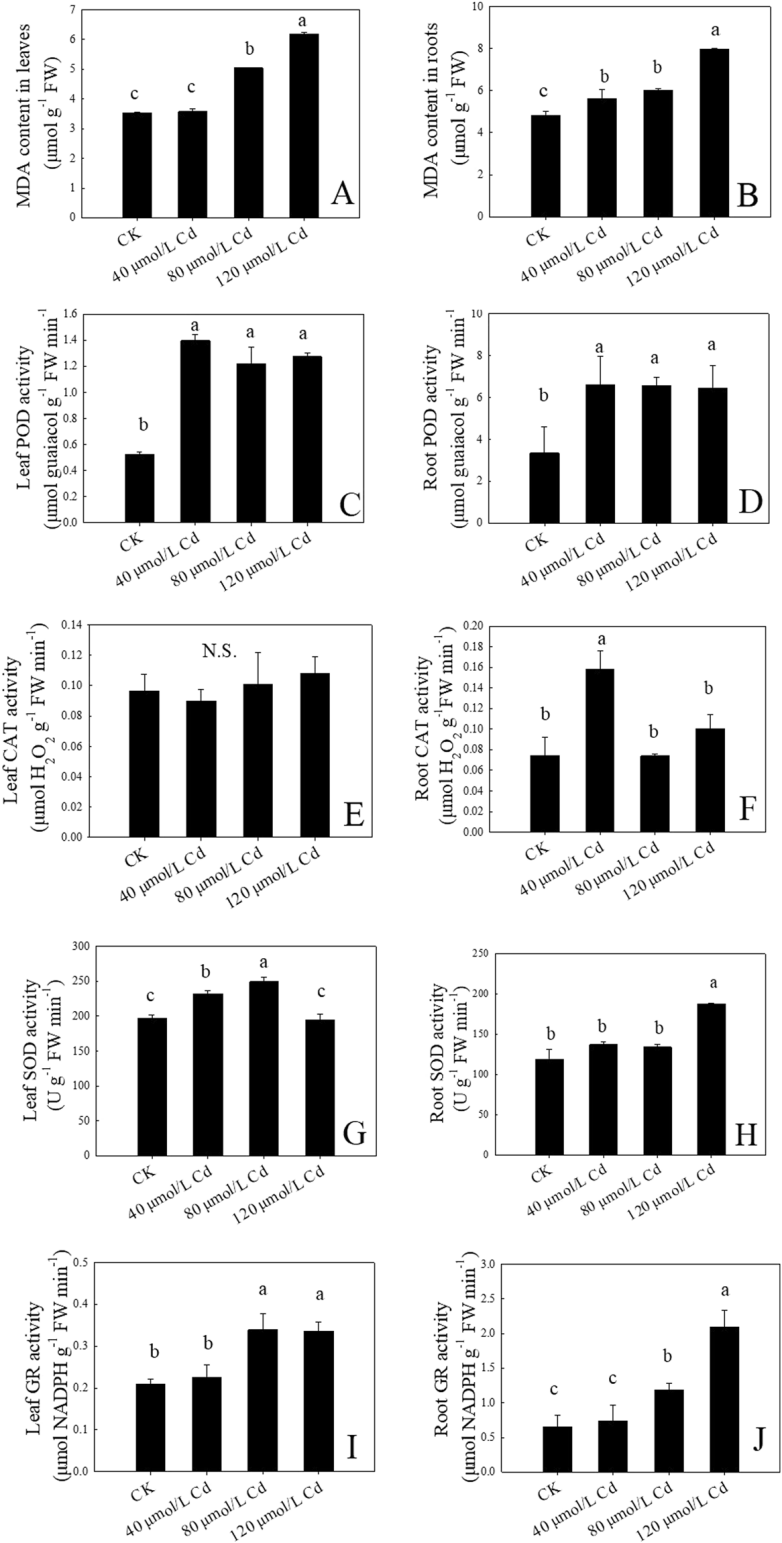
Physiological parameters of *C. bipinnatus* under different concentrations of Cd. A and B: the concentration of MDA; C and D: the POD activity; E and F: the CAT activity; G and H: the SOD activity; I and J: the GR activity. N.S., no difference in various treatment. Values were means ± standard deviation (n = 3); values followed by different lowercase letters show significant differences at *P* < 0.05.

In order to understand whether Cd induces biochemical damage, we investigated several biochemical indexes which are involved in oxidative stress. Compared with CK, Cd significantly increased the MDA concentrations in leaves (except of 40 μmol/L, Fig. 5A) and roots (Fig. 5B). Meanwhile, the POD activity in the leaves and roots were dramatically increased (Fig. 5C and D). Interestingly, Cd stresses did not affect the CAT activity in the leaves (Fig. 5E) and roots (except of 40 μmol/L, Fig. 5F). Except of 120 μmol/L Cd in the leaves, the SOD activity in the leaves and roots were increased by all three Cd treatments (Fig. 5G and H). Although 40 μmol/L Cd did not affect the GR activity, 80 and 120 μmol/L Cd significantly increased the GR activity in leaves and roots (Fig. 5I and J). These results indicated that lower Cd treatment (40 μmol/L) did not cause severe oxidative stresses, while higher Cd treatment (80–120 μmol/L) produced more oxidative damages.

### Transcriptome sequence and *de novo* assembly

Cd stresses changed the accumulation of some metal nutrients, the subcellular distribution of Cd, and the concentration and activity of some biochemical indexes. Meanwhile, some significantly changes were induced by 40 μmol/L Cd and no obvious toxic symptom was observed. Root samples treated with 40 μmol/L Cd was interestingly used to transcriptome analysis to reveal molecular response. 14.9 Gb nucleotides were generated (Table 2), which was deposited in the Sequence Read Archive (SRA) database with the accession numbers SRR3546768 and SRR3546769. 66,407 unigenes with the means length of 817 bp and N50 value of 1,344 bp were assembled. Among these assembled unigenes, the length of 18,491 unigenes (27.8% of all the unigenes) was more than 1,000 bp (Table 2). These results suggested that RNA sequencing and assembled unigenes had well quality and could be used for further transcriptome analysis.

**Table 2:** Overview of the reads and assembly.

Items	Number
Total nucleotides (nt)	14,940,933,000
Unigenes	66,407
Total length of unigenes (bp)	54,271,910
Mean length of unigenes (bp)	817
N50 length of unigenes (bp)	1,344
Length range more than 1000 bp	18,491

### Functional annotation and classification

41,674 (62.76%) unigenes were functionally annotated in at least one of the five databases: GO, KEGG, COG, Swissprot and NR (Table 3). 24,733 (37.24%) unigenes were not annotated in any public database. Among these annotated unigenes, 15,481 unigenes were classified into 25 COG categories. In detail, the major group of COG was ‘general functions prediction only’, followed by ‘translation, ribosomal structure and biogenesis’, ‘transcription’ and ‘replication, recombination and repair’ (SFig. 1). 24,639 unigenes were classified into three major categories of GO classification. ‘Cell’, ‘organelle part’ and ‘cell part’ represented the largest proportion in the cellular component category, while ‘catalytic activity’ and ‘cell part’ represented the most abundant categories in molecular function category. Moreover, the most abundant categories were ‘metabolic process’, ‘cellular process’ and ‘single-organism process’ in biological process category (SFig. 2). A total of 18,496 unigenes were annotated in the KEGG database and were classified into 128 KEGG pathways (STable 2). Briefly, ‘ribosome’ pathway (ko03010) contains the most abundant unigenes, followed by ‘carbon metabolism’ (ko01200), ‘biosynthesis of amino acids’ (ko01230), and ‘protein processing in endoplasmic reticulum’ (ko04141). All sequences and functional information were deposited in the NCBI Transcriptome Shotgun Assembly database with accession number GEZQ00000000.

**Table 3:** Result of unigne annotation.

Annotation Database	Annotated Number	The percentage of annotated unignenes in total unigenes (%)
GO Annotation	24,639	37.10
KEGG Annotation	18,496	27.85
COG Annotation	15481	23.31
Swissprot Annotation	27,069	40.76
NR Annotation	41,145	61.96
Annotated in at least one database	41,674	62.76

### Identification and functional characterization of differentially expressed genes in response to Cd stress

In present study, a log-fold expression change (log_2_FC) >2 or <−2 with P values <0.0005 and FDR <0.001 was used to determine the differentially expressed genes (DEGs). Compared with CK, 2,658 unigenes including 1,292 up-regulated unigenes and 1,366 down-regulated unigenes were induced by Cd (SFig. 3). Based on GO annotation, a total of 2,460 DEGs were classified into three GO major categories (SFigure 4A–C). Moreover, a total of 1,884 DEGs were annotated in KEGG pathway (STable 3).

### Noteworthy DEGs and metabolic pathways related to Cd uptake, transportation and detoxification

**Figure 6:**
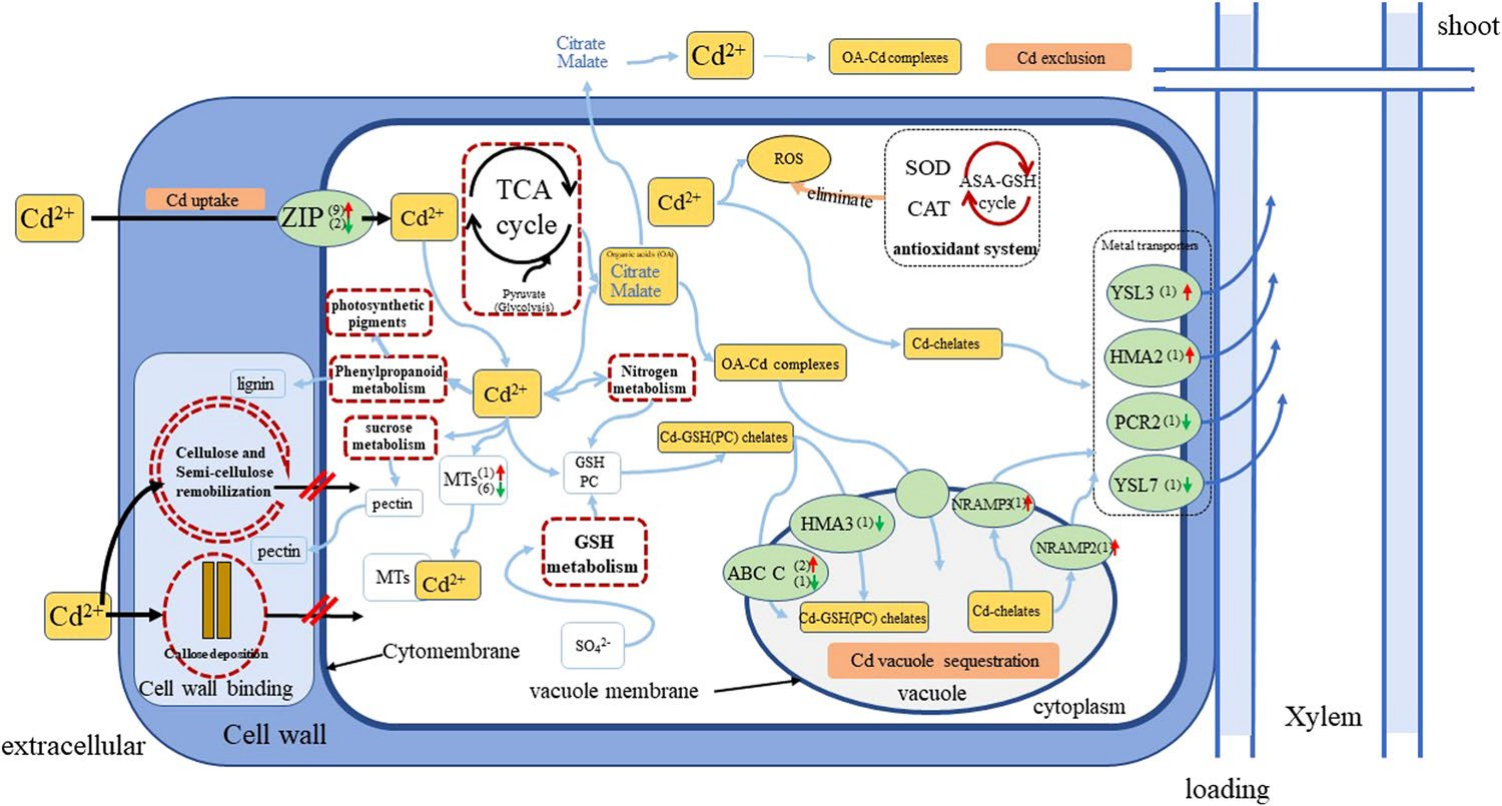
The presumed transcriptional network related to Cd uptake, translocation, and detoxification in *C. bipinnatus* root. The red arrows represent up-regulated genes, while the green arrows represent down-regulated genes. The dotted red boxes represent noteworthy mechanism pathways.

As shown in Fig. 6, a network associated to Cd uptake, transport, translocation, and detoxification were aggregated. Cd in extracellular could be obstructed by cell wall structures for toxicity reduction, while part of Cd would be transported by some metal transporters. When Cd entered into the *C. bipinnatus* cells, various metabolic processes could be induced for Cd detoxification. GSH, MTs and organic acid would be bound with Cd and then sequestrated into vacuoles. Cd also induced unigenes of antioxidant enzymes for oxidative defense. Finally, some Cd-chelates would be uploaded into xylem for transportation by some metal transporters.

Briefly, this network was mainly composited by the several following processes. Firstly, total 49 metal transporters (34 up-regulated and 15 down-regulated) including zinc transporters (ZIPs), ATP-binding cassette (ABC) family members, heavy-metal ATPases (HMAs) like *HMA3*, *HMA2*, the family of natural resistance-associated macrophage protein (NRAMP) members such as *NRAMP2*, *NRAMP3*, yellow stripe-like (YSL) family members, and plant cadmium resistance protein *PCR2* were observed (Table 4), suggesting that these metal transporters might participate in Cd uptake, transport and translocation. 29 DEGs (14 up-regulated and 15 down-regulated) involved in sulfate and GSH metabolism such as gene encoding adenylyl-sulfate kinase, sulfate adenylyl-transferase, serine acetyltransferase, adenylyl-sulfate reductase, sulfite reductase, serine acetyl-transferase, cysteine synthase, ornithine decarboxylase, spermidine synthase, glutathione S-transferase, and γ-glutamyl-transpeptidase 3 were regulated by Cd, suggesting the potential biosynthesis of GSH and PCs and chelation of Cd (Table 4, SFig. 5). 7 DEGs (1 up-regulated and 6 down-regulated) were metal chelates such as metallothioneins (MTs), suggesting MTs also play role in Cd chelation (Table 4). 30 DEGs (13 up-regulated and 17 down-regulated) were involved in cell wall metabolism, such as UDP-glucose 6-dehydrogenase1 (*UGDH1)*, UDP-glucose pyrophosphorylase2 (*UGP2*), glucose-6-phosphate isomerase (*GPI*), sucrose synthase, pectinesterase, UDP-glucuronate-4-epimerase, fructokinase, beta-fructofuranosidase, xyloglucan endotransglucosylase/hydrolase protein, expansin, glucan 1,3-alpha-glucosidase, cellulose synthase, callose synthase, and laccase, suggesting the compositions of cell wall were changed, mainly focused on pectin, callose and cellulose (Table 4). In addition, 12 DEGs (1 up-regulated and 11 down-regulated) participated in phenylpropanoid metabolism were regulated by Cd (Table 4). 17 DEGs (9 up-regulated and 8 down-regulated) were participated in tricarboxylic acid cycle (TCA) pathway, which potentially enhanced glucose metabolism and produced more organic acids such as citrate and malate for Cd binding (Table 4, SFig. 6). 11 DEGs (4 up-regulated and 7 down-regulated) were related to nitrogen metabolism, including genes encoding high-affinity nitrate transporter, nitrate reductase, ferrddoxin-nitrite reductase, glutamine synthetase, and glutamate synthase [NADPH/NADH] (Table 4). Moreover, 14 DEGs (4 up-regulated and 10 down-regulated) involved in antioxidant system, like superoxide dismutase, catalase isozyme, phospholipid hydroperoxide glutathione peroxidase, monodehydroascorbate reductase, and ascorbate peroxidase were differentially regulated by Cd (Table 4

**Table 4:** Noteworthy DEGs and metabolic pathways related to Cd uptake, transportation and detoxification.

Unigenes ID	Log_2_FC	Description
**Metal transporter**		
c69105_c0	9.44	ABC transporter A family member 7
c84507_c0	8.64	Zinc transporter 5
c65267_c0	8.04	ABC transporter B family member 4
c39965_c0	7.91	ABC transporter G family member 17
c83650_c0	7.18	ABC transporter G family member 43
c70109_c0	6.61	Zinc transporter 7
c54092_c1	6.42	Zinc transporter ZTP29
c10112_c0	6.20	ABC transporter F family member 4
c77338_c0	6.15	ABC transporter C family member 2
c59065_c0	5.97	Zinc transporter ZTP29
c65853_c2	5.84	ABC transporter F family member 1
c80839_c1	5.82	ABC transporter B family member 21
c32003_c0	5.77	ABC transporter F family member 4
c82788_c0	5.36	ABC transporter B family member 11
c37195_c0	5.16	Metal transporter Nramp3
c87790_c0	5.09	Metal transporter Nramp2
c35172_c0	5.04	ABC transporter G family member 22
c37809_c0	5.01	ABC transporter A family member 1
c56776_c0	4.72	Cadmium/zinc-transporting ATPase HMA2
c90431_c0	4.69	Zinc transporter 3
c66824_c0	4.69	Zinc transporter 1
c77338_c1	4.26	ABC transporter C family member 14
c59283_c0	3.60	Zinc transporter 5
c60866_c0	3.30	ABC transporter G family member 14
c76227_c0	3.16	ABC transporter A family member 2
c81234_c0	3.13	Zinc transporter 4
c73636_c0	2.89	Zinc transporter 4
c78055_c0	2.74	ABC transporter G family member 1
c60915_c0	2.59	ABC transporter B family member 11
c60185_c0	2.59	ABC transporter F family member 4
c68969_c0	2.52	Metal-nicotianamine transporter YSL3
c62568_c0	2.51	ABC transporter G family member 14
c82493_c0	2.29	ABC transporter G family member 16
c81263_c0	2.19	ABC transporter G family member 22
c23361_c0	−2.43	ABC transporter F family member 4
c74639_c0	−2.50	ABC transporter F family member 4
c68390_c0	−2.55	Cadmium/zinc-transporting ATPase HMA3
c28965_c0	−2.67	Metal-nicotianamine transporter YSL7
c66361_c0	−2.76	Metal-nicotianamine transporter YSL14
c57525_c0	−2.79	ABC transporter F family member 1
c27812_c0	−3.28	ABC transporter F family member 4
c67525_c0	−3.75	ABC transporter F family member 3
c97488_c0	−4.38	ABC transporter B family member 4
c3952_c0	−4.63	ABC transporter B family member 1
c98738_c0	−4.78	ABC transporter C family member 2
c85857_c0	−5.30	Zinc transporter 8
c53195_c0	−5.34	Plant cadmium resistance protein 2 PCR2
c27275_c0	−5.68	Zinc transporter 5
c89759_c0	−6.01	ABC transporter G family member 40
**Sulfate, GSH metabolism**		
c85588_c0	−6.46	glutathione S-transferase
c57929_c0	−6.28	Monodehydroascorbate reductase
c85420_c0	−6.23	Glutathione S-transferase F9
c85507_c0	−6.18	Glutathione S-transferase U8
c66368_c0	−6.09	L-ascorbate peroxidase 1
c85932_c0	−6.07	Glutathione S-transferase F6
c88021_c0	−5.71	glutathione S-transferase
c92596_c0	−5.71	Cysteine synthase
c87506_c0	−5.71	Glutathione S-transferase U17
c32193_c0	−5.39	glutathione S-transferase parC
c77199_c0	−5.06	L-ascorbate peroxidase 2
c94817_c0	−4.84	Glutathione S-transferase U17
c69512_c0	−2.84	Ornithine decarboxylase
c73219_c0	−2.53	Ornithine decarboxylase
c66300_c0	−2.43	Ornithine decarboxylase
c67901_c1	2.37	γ-glutamyl-transpeptidase 3
c80321_c1	2.64	γ-glutamyl-transpeptidase 3
c60771_c0	2.68	Adenylyl-sulfate kinase 3
c54934_c0	3.59	Sulfate adenylyl-transferase
c56904_c0	4.02	Monodehydroascorbate reductase
c40445_c0	4.69	Spermidine synthase 1
c88608_c0	4.79	L-ascorbate peroxidase 7
c67547_c0	4.86	adenylyl-sulfate reductase 3
c65681_c0	4.97	Cysteine synthase
c31632_c0	5.15	Serine acetyltransferase 5
c51737_c1	5.88	Glutathione S-transferase L2
c69922_c0	5.99	Glutathione S-transferase F9
c52639_c0	6.36	Glutathione S-transferase F13
c27387_c0	6.85	Sulfite reductase
**Metallothioneins(MTs)**		
c83366_c0	−9.45	Metallothionein-like protein type 2
c83540_c0	−8.79	Metallothionein-like protein type 3
c39380_c0	−8.50	Metallothionein-like protein type 3
c84110_c0	−7.91	Metallothionein-like protein type 2
c10641_c0	−7.39	Metallothionein-like protein 1
c85025_c0	−5.39	Metallothionein-like protein type 2
c25697_c0	5.27	Metallothionein-like protein type 2
**Phenylpropanoid metabolism**		
c84209_c0	−7.69	Peroxidase 42
c62400_c0	−7.33	Peroxidase 4
c52362_c0	−6.53	Caffeic acid 3-O-methyltransferase COMT
c88098_c0	−6.44	Trans-cinnamate 4-monooxygenase
c84029_c0	−6.41	Peroxidase 42
c87932_c0	−6.09	Peroxidase 15
c86814_c0	−5.39	Phenylalanine ammonia-lyase PAL
c90791_c0	−5.14	Cinnamyl alcohol dehydrogenase 1 CAD1
c90727_c0	−4.84	Trans-cinnamate 4-monooxygenase
c86953_c0	−4.71	Caffeic acid 3-O-methyltransferase COMT
c98254_c0	−4.47	Caffeic acid 3-O-methyltransferase COMT
c79992_c0	2.10	4-coumarate–CoA ligase-like 4CL
**Cell wall metabolism**		
c52549_c0	6.75	Beta-fructofuranosidase
c55905_c0	−5.85	Fructokinase-4
c22672_c0	4.75	Glucose-6-phosphate isomerase GPI
c63434_c0	3.15	Pectinesterase 2
c88310_c0	−6.09	Pectinesterase 3
c92007_c0	−5.03	Pectinesterase U1
c63222_c0	−7.80	Sucrose synthase 1
c25867_c0	−5.98	Sucrose synthase 2
c88935_c0	−5.25	Sucrose synthase 2
c87544_c0	−5.56	Sucrose synthase 3
c86190_c0	−4.97	Sucrose synthase 3
c53972_c0	−6.18	UDP-glucose 6-dehydrogenase 1 UGDH1
c43360_c0	6.03	UDP-glucose pyrophosphorylase UGP2
c86968_c0	−5.92	UDP-glucuronate 4-epimerase 1
c36225_c0	−6.37	UDP-glucuronate 4-epimerase 4
c32187_c0	−5.60	UDP-glucuronate 4-epimerase 6
c86761_c0	−5.64	UDP-glucuronate 4-epimerase 6
c84524_c0	2.21	Xyloglucan endotransglucosylase/hydrolase protein 23
c84832_c0	2.87	Xyloglucan endotransglucosylase/hydrolase protein 32
c87800_c0	−5.09	Xyloglucan endotransglucosylase/hydrolase protein 6
c87242_c0	−5.09	Xyloglucan endotransglucosylase/hydrolase protein B
c64115_c0	2.67	Xyloglucan endotransglucosylase/hydrolase protein 32
c27718_c0	−5.75	Xyloglucan endotransglucosylase/hydrolase protein 9 (Precursor)
c75386_c0	5.02	Callose synthase 7
c80747_c0	2.55	Cellulose synthase-like protein G3
c49538_c0	2.97	Expansin-A1
c50143_c0	2.46	Expansin-A10
c63814_c0	5.05	Glucan 1,3-alpha-glucosidase
c66127_c0	3.21	Laccase-12
**TCA cycle**		
c87855_c0	−6.01	Aconitate hydratase ACO
c88735_c0	5.48	ATP-citrate synthase alpha chain protein 1 ACLA
c89044_c0	−6.62	Dihydrolipoyl dehydrogenase 1 LPD1
c91756_c0	3.72	Dihydrolipoyl dehydrogenase 1 LPD1
c33952_c0	6.55	Dihydrolipoyl dehydrogenase LPD
c71205_c0	2.53	Dihydrolipoyllysine-residue acetyltransferase DLAT
c87987_c0	3.98	Isocitrate dehydrogenase IDH3
c56559_c0	−5.92	Malate dehydrogenase MDH
c59906_c0	−5.52	Malate dehydrogenase MDH
c99710_c0	−4.84	Malate dehydrogenase MDH
c61978_c0	−4.71	Malate dehydrogenase MDH
c91986_c0	−5.09	Succinate dehydrogenase
c23134_c0	−5.82	Malate dehydrogenase1 MDH1
c52564_c0	3.59	Pyruvate dehydrogenase PDHA
c85739_c0	4.49	Pyruvate dehydrogenase PDHA
c87218_c0	4.93	Pyruvate dehydrogenase PDHA
c57876_c0	5.79	Succinate dehydrogenase [ubiquinone] iron-sulfur subunit 1 SDHB
**nitrogen metabolism**		
c86348_c0	−6.61	Glutamate synthase [NADH]
c85579_c0	−6.18	Glutamine synthetase
c86171_c0	−5.26	Glutamine synthetase
c39565_c0	−5.17	Glutamine synthetase
c88704_c0	−5.09	Nitrate reductase [NADH]
c91005_c0	−4.97	Glutamate synthase 1 [NADH]
c24081_c0	−4.47	Glutamine synthetase
c72948_c0	3.64	Ferredoxin–nitrite reductase
c66268_c0	5.30	High-affinity nitrate transporter 2.2
c53175_c0	5.54	Glutamine synthetase
c87775_c0	5.70	Nitrate reductase [NADH]
**antioxidant system**		
c85366.c0	−7.03	Superoxide dismutase [Cu-Zn]
c88413.c0	−4.55	Superoxide dismutase [Cu-Zn]
c58063.c0	−5.60	Superoxide dismutase [Mn]
c86851.c0	−4.93	Catalase isozyme 1
c85554.c0	−6.15	Catalase isozyme 2
c88142.c0	−4.63	Catalase isozyme 2
c86608.c0	−4.38	Catalase-2
c31799_c0	5.07	Phospholipid hydroperoxide glutathione peroxidase 1
c33214_c0	4.83	Probable phospholipid hydroperoxide glutathione peroxidase 6
c56904_c0	4.02	Monodehydroascorbate reductase
c57929_c0	−6.28	Monodehydroascorbate reductase
c66368_c0	−6.09	L-ascorbate peroxidase 1
c77199_c0	−5.06	L-ascorbate peroxidase 2
c88608_c0	4.79	L-ascorbate peroxidase 7

).

### RT-qPCR validation

**Figure 7:**
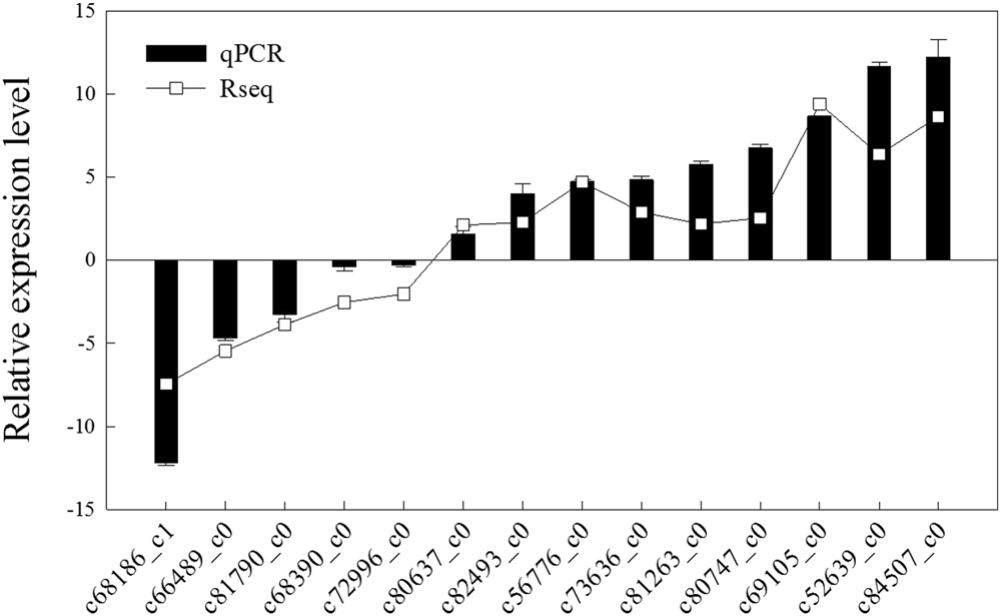
Quantitative RT-PCR of selected DEGs under control and Cd treatment in *C. bipinnatus*. The black bar represents the result of Rseq calculated by FPKM. The black bar with standard deviation represents the relative expression level determined by qPCR analysis.

To confirm the differential expression profiles of DEGs identified from RNA-Seq analysis, a total of 14 candidate DEGs were randomly selected from RNA-Seq and their expression levels in CK and Cd were examined by quantitative RT-PCR. As expected, the expression pattern of those unigenes obtained from qRT-PCR was similar with the differential expressions from RNA-Seq (Fig. 7).

## Discussion

Under Cd stress, if the plants accumulate more than 100 μg/g Cd in dry aerial tissue, the value of TF is more than 1, and with normal growth, these plants are recommended as Cd hyper-accumulators^37–39^. However, most plants exhibit Cd toxicity when the leaves accumulate more than 5–10 μg/g Cd^40^. In the present study, *C. bipinnatus* accumulated 60.36 ±  2.17 μg/g Cd in the leaves, 321.15 ± 16.04 μg/g Cd in the stems, and 576.65 ± 41.48 μg/g Cd in the roots under 40 μmol/L Cd treatment without showing obvious toxic symptoms (Table 1 and Fig. 1), indicated that *C. bipinnatus* has strong tolerance to Cd. Although the TF values of *C. bipinnatus* were less than 1 (ranged from 0.66–0.79), the Cd concentrations of leaves and stems individually reached to 60.36 and 321.15 μg/g. Thus, *C. bipinnatus* should be a Cd accumulator, which would be potentially used for phytoremediation under mild Cd stress. However, the mechanism of strong tolerance and high Cd accumulation of *C. bipinnatus* was unknown. The physiological parameters and transcriptome analysis would help us revealing the mechanism.

Normally, Cd was intake, translocated and accumulated using other metal transporters, such as Zn, Fe and Cu^41^. Although *ZIP* genes mainly transport Zn, some *ZIPs* participate in Cd transport in *Arabidopsis* and *Thlaspi caerulescens* ^42^. We found that Cd up-regulated *ZIP1*, *ZIP3*, *ZIP5*, *ZIP7*, and *ZTP29* (Table 4), suggesting that these genes were involved in Cd and Zn transport, thus the Zn concentrations in roots were reduced (Fig. 4A). *HMA2*, a Cd/Zn transporter, loads Cd/Zn into xylem^43,44^. The increased expression level of *HMA2* in *Arabidopsis* ^45^, rice^44^, and barley^43^ induced Cd or Zn xylem uploading for translocation to the shoot. *HMA3*, another P-type ATPase gene, segregates Cd or Zn into the root vacuolar to limit the Cd xylem loading^46,47^. Down-regulation of *HMA3* resulted in a decreased concentration of Cd in the root^48^. Certainly, when *HMA3* is localized at the leaf vacuolar, it also transports Cd into the leaf vacuolar, finally producing Cd hyper-accumulator of *Thlaspi caerulescens* ^16^. Therefore, in the present study, up-regulation of *HMA2* and down-regulation of *HMA3* not only implied most of Cd was uploaded to aerial tissues, which resulted in high root-shoot translocation (Table 1), but also suggested a certain amount of Zn was uploaded to shoots, so that higher Zn concentrations accumulated in aerial tissues under Cd treatment (Fig. 4B and C). NRAMP (nature resistance associated with microphage) family members display poor selectivity towards divalent mental cations, which are responsible for heavy metal ions uptake and transport^49^. Previous studies have found that *NRAMP1*, *NRAMP3*, *NRAMP4* and *NRAMP6* transport Fe and Cd^49–52^. *NRAMP2* and *NRAMP3* involved in metal efflux from the vacuole^49,53,54^. In *Arabidopsis*, increased expression levels of *NRAMP3* result in an increased metal output from the vacuole^55^. Two NRAMP family genes (*NRAMP2* and *NRAMP3*) were both up-regulated by Cd in our research, suggesting that they involved in Fe or Cd efflux from vacuole, thereby leading to high accumulation of Fe in aerial tissues (Fig. 4H and I). Higher Fe concentration in shoots alleviated the Cd toxicity in *Arabidopsis* ^56^. Therefore, the increased concentration of Fe in leaves of *C. bipinnatus* may also be associated with the detoxification of the plant. Additionally, *NRAMP2* and *NRAMP3* may also have a role in Cd transport processes. *YSL* genes participate in Fe-nicotinamide (Fe-NA) complex root-to-shoot transport^57^. Cd mediated the expression of *YSL3* and *YSL7*, suggesting Cd affected Fe transport in the plant. Previous study also found that *YSL3* in *Solanum nigrum* and *YSL7* in *Brassica juncea* were induced by Cd as well^58,59^. However, it remains unknown whether YSL family genes are involved in transport of Cd-NA complexes transport, and further study must be conducted to analyze the function of YSL genes under Cd stress.

The subcellular distribution of Cd in the root is associated with the accumulation, translocation, and detoxification of Cd^60^. Cd bound in the cell wall fraction is an important mechanism for Cd tolerance^61–63^. A large part of Cd was bunded in root cell wall of *C. bipinnatus*, especially at high Cd concentration treatment (Fig. 3). Cell wall is comprised of polysaccharide (including cellulose, semi-cellulose and pectate) and protein^64,65^. Containing abundant hydroxyl (OH-) for metal binding^66^, cellulose and semi-cellulose are both essential components of primary and secondary cell walls of higher plants^31,67^. In addition, cellulose synthase plays important role in cellulose formation, while xyloglucan endotransglucosylase (XTH) are involved in cell wall extension by cutting loosened xyloglucan strands and integrating new xyloglucans into the cell walls^68^. Present study found that the genes encoding cellulose synthase and XTH are up-regulated by Cd, suggesting the synthase and remobilization of cellulose and semi-cellulose play critical role in Cd binding. Moreover, the existence of pectin enhances the binding capacity of cell wall^69,70^. The synthase of pectin is associated with glucose metabolism. Several DEGs involved in *UGP2*, *GPI*, beta-fructofuranosidase and pectinesterase were up-regulated by Cd treatment (Table 4, SFig. 7), suggesting that Cd induce formation of pectin, thereby enhancing the capacity of Cd accumulation in cell walls, resulting high Cd tolerance of *C. bipinnatus*. Moreover, callose functions as a mechanical barrier to prevent ions penetration^52,71–73^, and the multi-copper-containing glycoprotein laccases involved in cell wall lignification^74^. The unigenes encoding callose synthase and laccase were up-regulated by Cd treatments in our study, implied that *C. bipinnatus* accumulated callose deposition and enhance cell wall lignification in root to prevent Cd entering the protoplasts under Cd stress. These results indicated that cell wall obstruction is one of important detoxification mechanism in *C. bipinnatus*.

After Cd entered into cytoplasm, it would be bound with metal chelates and then sequestrated into vacuoles to reduce their toxicity. The result of Cd subcellular distribution demonstrated that a large proportion of Cd was found in soluble fractions, suggesting the vacuole was the predominant detoxification sink for Cd in *C. bipinnatus* root. Vacuole possesses abundant sulphur-rich peptides such as GSH and PCs^75–77^, and organic acids^78^, which binds heavy metals and decreases their migration to reduce toxicity. Interestingly, unigenes involved in glutathione (GSH) metabolism, including serine acetyltransferase, cysteine synthase, ornithine decarboxylase, spermidine synthase, glutathione S transferase, γ-glutamyltranspeptidase, and unigenes from *ABCC* family were up-regulated under Cd treatment (Table 4). Meanwhile, *ABCC1*, *ABCC2* and *ABCC3* are major vacuolar PC-Cd transporters in other plants^75,79^, which were also up-regulated. Thus, Cd in cytoplasm turned into GSH (PC)-toxic compounds, finally transported by *ABCC* transporters without displaying cytotoxic to plant cell^80–82^. Vacuole sequestration of Cd also plays main role in *Phytolacca Americana* ^83^ and *Arachis hypogaea* ^84^. Moreover, GSH is also one of important antioxidants in plants. AsA -GSH cycle system can be able to eliminate ROS in many plants. Genes encoding enzymes involved in AsA-GSH cycle like glutathione peroxidase, monodehydroascorbate reductase and L-ascorbate peroxidase were up-regulated by Cd. Similarly, the activity of GR under Cd stress significantly increased compared with CK. These results indicated that enhancement of AsA-GSH cycle improved Cd tolerance of *C. bipinnatus*.

MDA is the product of lipid peroxidation, and its concentration reflects the degree of oxidative damage. In our study, 40 μmol/L Cd did not increase the MDA concentrations in leaves, while MDA concentration increased in roots of *C. bipinnatus* with higher accumulation of Cd (Fig. 5A and B), indicating that *C. bipinnatus* had strong tolerance under lower Cd but suffered cellular oxidative stress at higher Cd. Activities of antioxidant enzymes are induced by oxidative stress, and increased antioxidant level prevent oxidative damages^85^. Previous studies illustrated their functions in scavenging ROS in plants^86,87^. The enzyme SOD alters O_2_^−^ to H_2_O_2_ and oxygen^88^. CATs convert H_2_O_2_ to water and molecular oxygen, while PODs have a more elevated affinity to H_2_O_2_ than CATs^89^. The coordination between different enzymes can alleviate oxidative stress in the plant. In our study, compared with CK, the activity of POD and SOD in leaves were significantly increased under 40 μmol/L Cd treatment (Fig. 5C and G), while other two enzymes did not show significant changes (Fig. 5E and I), suggested that the activities of SOD and POD possessed sufficient capacity to scavenging ROS under lower Cd treatment. Therefore, the MDA concentration did not increase under 40 μmol/L Cd. However, generation of ROS were increased with the increased of Cd concentrations, exceeding the limits of POD and SOD scavenging ability (Fig. 5I). Meanwhile, the GR activity increased, complementing the ability of ROS scavenging. In addition, two unignenes encoding *peroxidase* were up-regulated under Cd treatment. These results demonstrated that antioxidative enzymes indeed play an important role in Cd detoxification and enhance tolerance of *C. bipinnatus* under adverse environment.

Summary*, C. bipinnatus* was recommended as a “Cd-accumulator” that would be potentially used for phytoremediation under mild Cd stress. Subcellular distribution of Cd displayed different detoxification mechanisms under different levels of Cd stress. *C. bipinnatus* initiated diverse defense and detoxify response to keep strong tolerance when treated with Cd stress. RNA-Seq analysis revealed that *ZIPs*, *NRAMPs*, *HMAs*, and *ABC* transporters were involved in Cd uptake, translocation and accumulation. Meanwhile, several processes such as cell wall biosynthesis, glutathione (GSH) metabolism, TCA cycle and the antioxidant system probably played critical roles in cell wall binding, vacuole sequestration and detoxification.

## Materials and Methods

### Plant culture and Cd treatment

Cosmos seeds (*Cosmos bipinnatus* Cav.) were sterilized with 2% NaClO for 20 min then rinsed with deionized water. The sterilized seeds were germinated on clean sand at 25 °C. After 2 weeks, the uniform seedlings were transplanted into plastic pots with 2.5 L half-strength Hoagland nutrient (60 plants per pot, pH 6.5) for 7 days and this was then replaced with full Hoagland nutrient solution. The plastic pots were randomly divided into 4 groups, each in triplicate. The four groups were treated with control, 40, 80, and 120 μmol/L CdCl_2_, respectively. All plants grew in a growth chamber with a daily temperature of 25 °C, a relative humidity of 70% and a photon flux density of 500 μmol/m^2^·S. Leaf, stem and root samples of all the treatments were collected on the 9th day. The root samples from 10 plants (10 plants per replicate, three biologic repeats) were collected and rapidly frozen in liquid nitrogen and then stored at −80 °C for RNA extraction.

### Phenotype characterization

On the 9th day after treatment, the plant height and root length were measured (12 plants per biological replicate, three biologic repeats). The fresh weight of root, stem and leaf were also weighted. After weighing, all tissues were then dried at 80 °C for two days for dry weight calculation and metal concentration measurement.

### Measurement of metal concentrations and calculation of translocation factor (TF)

The concentration of Cd, Zn, Fe, and Ca was measured as described by Wang *et al*.^90^ with some modifications^90^. Briefly, approximately 0.2 g dried plant samples were ground into powder which was digested at 320 °C with mixed acid [HNO_3_ + HClO_4_ (4:1, v/v)]. The concentration of Cd, Zn, Fe, and Ca in the digestions was detected by FAAS (flame atomic absorbance spectrometry, Shimadzu AA-6300, Kyoto, Japan). The limit for Cd, Zn, Fe, and Ca detection was 0.02 mg/L and a reference standard solution was purchased from Fisher Scientific Ltd. (China). The translocation factor was calculated as described by Li *et al*.^91^.

### Malondialdehyde (MDA) determination

MDA in the roots and leaves was determined according to the method of Wang and Jin (2005) with some modifications^92^. Briefly, 0.2 g of fresh sample was homogenized in 6 mL 20% trichloroacetic acid (TCA) and centrifuged at 4000 r/min for 10 min at 4 °C. The mixture containing 2 mL of the supernatant and 2 mL of 0.6% thiobarbituric acid (TBA) in 10% TCA was incubated at 95 °C for 30 min and cooled immediately, then centrifuged at 4000 r/min for 5 min. The absorbance of the supernatant was recorded at 450, 532, and 600 nm. The concentration of MDA was calculated according to the following equation:

$${C}_{MDA}=6.45({A}_{532}-{A}_{600})-0.56{A}_{450}$$

### Determination of four enzymatic activities

Approximately 0.5 g of fresh leaf or root sample was homogenized in 5 mL of pre-cooled 50 mmol/L Tris-HCl buffer (pH 7.0) containing 1 mmol/L EDTA, 1 mmol/L DTT, 5 mmol/L MgCl_2_, 1 mmol/L AsA, and 1 mmol/L GSH^93^. Then the homogenate was centrifuged at 12000 r/min for 20 min at 4 °C and the extract was used for the enzyme assay.

The superoxide dismutase (SOD) activity was determined according the method described earlier^94^. The reaction mixture consisted of 50 mmol/L Tris-HCl buffer (pH 7.8), 0.1 mmol/L EDTA, 0.1 mmol/L nitroblue tetrazolium (NBT), 13.37 mmol/L methionine, and 0.1 mmol/L riboflavin and enzyme extract. The reaction was initiated by adding the riboflavin. The mixture was first placed under light then transferred into darkness immediately and the absorbance recorded at 560 nm. One unit of SOD activity was defined as the amount of enzyme that inhibited 50% of NBT photoreduction.

The catalase (CAT) activity was assayed in a reaction mixture containing 2.9 mL 50 mmol/L Tris-HCl buffer (pH 7.0), 50 μL 750 mmol/L H_2_O_2_, and 50 μL enzyme extract as per the method of Aebi (1984)^95^. Activity was measured by following the decomposition of H_2_O_2_ at 240 nm.

The peroxidase (POD) activity was determined according to the guaiacol method^96^ with some modifications. The reaction mixture was 50 mmol/L Tris-HCl buffer (pH 7.0) containing 0.1 mmol/L EDTA, 10 mmol/L guaiacol, 5 mmol/L H_2_O_2_ and 100 μL enzyme extract. The reaction was initiated by adding the extract. Guaiacol oxidation was determined based on an increase in the absorbance at 470 nm. One unit of POD activity was expressed as units (μmol guaiacol decomposed per minute) per mg of fresh weight (FW).

The glutathione reductase (GR) activity was assayed as described by Foyer and Halliwell (1976) with some modifications^97^. The reaction mixture consisted of 450 μL of the enzyme extract, 2.34 mL 50 mmol/L Tris-HCl buffer (containing 0.1 mmol/L EDTA, 5 mmol/L MgCl_2_ pH 7.5), 60 μL 10 mmol/L NADPH and 150 μL 10 mmol/L oxidized glutathione (GSSG). The reaction was initiated by adding the extract, NADPH, and GSSG. The NADPH oxidation rate was determined by recording the decrease in absorbance at 340 nm. The GR activity was expressed as the amount of enzyme needed to oxidize 1 μmol of NADPH /min· mg FW.

### Subcellular distribution of Cd in the *C. bipinnatus* root

Cd subcellular distribution was determined according to Su *et al*.^84^ with some modifications^84^. The frozen root samples (1 g) were ground into powder with a pre-cold extraction buffer [50 mmol/L Tris-HCl buffer solution (pH 7.5), 250 mmol/L sucrose, 1.0 mmol/L DTE (C_4_H_10_O_2_S_2_) and 5.0 mmol/L ascorbic acid]. The homogenate was centrifuged at 4000 r/min for 15 min and the precipitate was designated as a cell wall fraction consisting mainly of cell walls and cell wall debris. The supernatant solution was further centrifuged at 16000 r/min for 45 min. The resultant deposit and supernatant solution were designated as the organelle-containing fraction and the soluble fraction, respectively. All fractions were dried and then digested in 5 mL HNO_3_. The Cd concentrations in the different fractions were analyzed by FAAS.

### RNA extraction

The total RNA of each root sample (CK, 40 μmol/L Cd treatment) was extracted by using the Quick RNA isolation Kit (Huayueyang Biotech Co., Ltd., Bejing, China) according to the instruction manual. RNase-free DNasel (TaKaRa Biotech Co., Ltd., Dalian, China) was used for removing residual DNA in the extracted RNA. The quality of the total RNA sample was measured by 1% agarose gels, and the concentrations of the total RNA samples were assayed with an Agilent 2011 Bioanalyzer (Agilent Technologies, Inc., Santa Clara, CA, USA).

### Library construction and illumina sequencing

High-quality RNA samples from *C. bipinnatus* root were prepared for cDNA library construction and sequencing. The mRNA was purified from total RNA using oligo (dT) magnetic beads and poly (A) tails. The RNA sequencing libraries were generated using the TruSeq RNA sample Prep Kit (Illumina, San Diego, CA) with multiplexing primers, according to the manual. The cDNA library was constructed with average inserts of 250 bp, with non-stranded library preparation. The QIAquick PCR extraction kit (Qiagen, Inc., Hilden, Germany) was used for cDNA purifying. The short cDNA fragments were subjected to end repair, adapter ligation, and agarose gel electrophoresis filtration. Subsequently, the appropriate fragments were selected as templates for PCR amplification. Sequencing was performed via a paired-end 125 cycle rapid run on 2 lanes of the Illumina HiSeq. 2500 system, generating pairs of reads of great quality as intended.

### Transcriptome assembly

Adapter-related and low-quality reads including ambiguous reads (‘N’), duplicated sequences were removed from the raw reads to obtain the clean reads. Trinity software (http://trinityrnaseq.sourceforge.net/) was used for the *de novo* assembled transcriptomes. In brief, the contigs were formed by combining the certain overlap length into long fragments without N (contigs) and then they were clustered using the TGICL software to produce unigines (without N) and finally the redundancies were removed to obtain non-redundant unigenes^98^.

### Unigene functional annotation

A series of databases and software were used for putative unigenes annotations. BLAST software^99^ was used to align the unigene with the NR^100^, Swiss-Prot^101^, GO^102^, COG^103^, and KEGG databases^104^ (E-value ≤ 1E^−5^) to retrieve protein functional annotations based on sequence similarity. The ESTScan software was used to decide the sequence direction of the unigenes that could not be aligned to any of the above databases^105^. Functional categories of putative unigenes were grouped using the GO and KEGG databases.

### Differential expression analysis

FPKM values were used to compare gene expression differences between the two samples. The DESeq package was used to obtain the base mean value for identifying DEGs. FDR ≤0.01 and the absolute values of log2 ratio ≥1 were set as the thresholds for the significance of the gene expression difference between the two samples.

### Real-time quantitative (qRT-PCR) validation of partial DEGs

qRT-PCR was performed in a 96-well plate with the CFX-96 real-time system (Bio-Rad, CA, USA). Each reaction of 15 μL contained 6.3 μL (30 ng/μL) cDNA, 0.6 μL (4 pmol/μL) for each forward and reverse primer, and 7.5 μL iQ SYBR Green Supermix (Bio-Rad, CA, USA). Each cDNA sample was amplified in triplicates. The PCR reaction conditions were 95 °C for 5 min, 39 cycles of 95 °C for 15 s, 56 °C for 30 s, and 72 °C for 10 s, followed by the generation of a dissociation curve by increasing the temperature starting from 65 °C to 95 °C to check for the specificity of amplification. *Actin* was used to standardize the transcript levels in each sample. The relative expression level was calculated with the 2^−△△CT^ formula^106^. The primers that were designed and used in the RT-qPCR analyses are shown in STable 1.

### Data analysis

The *SPSS version* 22.0 software was used for statistical analyses. The mean and standard deviation (SD) of three replicates were calculated. Duncan’s test was used to determine the significant differences between means (*p* < 0.05)^107^. Besides, the figures were drawn with Sigmaplot 12.5.

## Electronic supplementary material

Supplementary information
