# Supplementary material for: Physiological and transcriptome response to cadmium in cosmos (Cosmos bipinnatus Cav.) seedlings
Source: Sci Rep. 2017 Oct 31;7:14691. doi: 10.1038/s41598-017-14407-8 (PMC5665871; doi:10.1038/s41598-017-14407-8)
Supplement: Supplementary file 1 — Supplementary information [file 41598_2017_14407_MOESM1_ESM.pdf]

Physiological and transcriptome response to cadmium in cosmos (*Cosmos bipinnatus* Cav.) seedlings

Yujing Liu [1, a], Xiaofang Yu [1, a*], Yimei Feng [1], Chao Zhang [2], Chao Wang [3], Jian Zeng [4], Zhuo Huang [1], Houyang Kang [3], Xing Fan [3], Lina Sha [3], Haiqin Zhang [3], Yonghong Zhou[3], Suping Gao [1] and Qibing Chen [1]

[1] Landscape Architecture, Sichuan Agricultural University, Wenjiang 611130, Sichuan, China

[2] Industrial Crop Research Institute of Sichuan Academy of Agricultural Sciences, Qingbaijiang 610300, Sichuan, China

[3] Triticeae Research Institute, Sichuan Agricultural University, Wenjiang 611130, Sichuan, China

[4] College of Resources, Sichuan Agricultural University, Wenjiang 611130, Sichuan, China

[a] These authors contributed equally to this work

*** Correspondence:**

Corresponding Author:

xiaofangyu@sicau.edu.cn

STable 1 The qRT-PCR primers of 14 DEGs

Unigenes ID	Description	sense primer	anti-sense primer
c68186_c1	Ribulose bisphosphate carboxylase large chain	CCGTGAACAGCCAACCAT	ACAGAGAGCCGACAAGGAA
c66489_c0	UDP-glycosyltransferase 74B1	CCGAGTTTGAAGGCTTGGTTT	AGGTGGTGAATGGCATAAGGA
c81790_c0	Photosystem II CP47 reaction center protein	ACAGTTCAAGTGGCAGTTAGTG	CTCAGGTTCATCGTCATCATCAT
c68390_c0	Cadmium/zinc-transporting ATPase HMA3	TAGTGGATGATGAGGTGTTGGTA	TGGTGCTGTTGCCTTGAC
c72996_c0	L-ascorbate oxidase homolog	GTAGCGTTGAGCATCGTTGA	TTGGTTGAGGTTGAAGGAACTC
c80637_c0	Galactinol--sucrose galactosyltransferase	TGGTTGAGCATACGAATCTTGT	CCGCATCTTGAGCCACTT
c82493_c0	ABC transporter G family member 16	ACGCTGCTAAGACTGTCATTG	ACGATGCTACCGCTCTGT
c56776_c0	Cadmium/zinc-transporting ATPase HMA2	CCTGGCTGAATGGCTTGAA	TTGGTTGAGTTGGTGTCTTGTT
c73636_c0	Zinc transporter 4	CCGTGTGAGTGTGAATGTGAAT	GGCTGTGAAGGACAAGTGAG
c81263_c0	ABC transporter G family member 22	TCTGTCTCGGCTATGTCTTGT	AATCCCTCCCTCTTGTTCCTT
c80747_c0	Cellulose synthase-like protein G3	GGACCGAACACCGTATATTAAGG	CATTGCGAAGGCTGGAAGT
c69105_c0	ABC transporter A family member 7	CGCATCCACATATCAAGCACAA	CAGCAGTAGCAGGTCCAGTA
c52639_c0	Glutathione S-transferase F13	ATGCCTGCTTGGACAACTG	TGGTGGACAACTGGATGGA
c84507_c0	Zinc transporter 5	CTGCTTGGAATGGAACTGCTAT	GATGAGAATGGACCTGCCTTG
c68921_c0	Actin (reference gene)	AGTTCCCTCTTAAATCGGTCCTT	CTGCTGAATGTCGTTATGCTGTA

STable 2 KEGG pathway of all the unigenes

The first level classification	The second level classification	Pathway definition	pathway ID	Number of genes
Genetic information processing	Translation	Ribosome	ko03010	1321
Metabolism	Global and overview maps	Carbon metabolism	ko01200	857
Metabolism	Global and overview maps	Biosynthesis of amino acids	ko01230	811
Genetic information processing	Folding, Sorting and degradation	Protein processing in endoplasmic reticulum	ko04141	600
Genetic information processing	Transcription	Spliceosome	ko03040	498
Metabolism	Energy metabolism	Oxidative phosphorylation	ko00190	475
Genetic information processing	Translation	RNA transport	ko03013	474
Environmental information processing	Signal transduction	Plant hormone signal transduction	ko04075	416
Metabolism	Nucleotide metabolism	Purine metabolism	ko00230	392
Metabolism	Carbohydrate metabolism	Starch and sucrose metabolism	ko00500	391
Metabolism	Carbohydrate metabolism	Glycolysis / Gluconeogenesis	ko00010	361
Cellular processes	Transport and catabolism	Endocytosis	ko04144	357
Genetic information processing	Translation	mRNA surveillance pathway	ko03015	302
Metabolism	Carbohydrate metabolism	Pyruvate metabolism	ko00620	301
Cellular processes	Transport and catabolism	Phagosome	ko04145	296
Genetic information processing	Folding, Sorting and degradation	Ubiquitin mediated proteolysis	ko04120	292
Metabolism	Energy metabolism	Carbon fixation in photosynthetic organisms	ko00710	291
Metabolism	Nucleotide metabolism	Pyrimidine metabolism	ko00240	287
Genetic information processing	Folding, Sorting and degradation	RNA degradation	ko03018	284

Metabolism	Global and overview maps	Fatty acid metabolism	ko01212	281
Metabolism	Amino acid metabolism	Cysteine and methionine metabolism	ko00270	270
Metabolism	Carbohydrate metabolism	Amino sugar and nucleotide sugar metabolism	ko00520	252
Metabolism	Biosynthesis of other secondary metabolites	Phenylpropanoid biosynthesis	ko00940	251
Metabolism	Energy metabolism	Photosynthesis	ko00195	248
Metabolism	Amino acid metabolism	Glycine, serine and threonine metabolism	ko00260	246
Metabolism	Metabolism of other amino acids	Glutathione metabolism	ko00480	241
Metabolism	Amino acid metabolism	Arginine and proline metabolism	ko00330	236
Organismal systems	Environmental adaptation	Plant-pathogen interaction	ko04626	235
Genetic information processing	Translation	Ribosome biogenesis in eukaryotes	ko03008	234
Metabolism	Carbohydrate metabolism	Glyoxylate and dicarboxylate metabolism	ko00630	232
Genetic information processing	Folding, Sorting and degradation	Proteasome	ko03050	221
Cellular processes	Transport and catabolism	Peroxisome	ko04146	216
Metabolism	Carbohydrate metabolism	Citrate cycle (TCA cycle)	ko00020	211
Metabolism	Amino acid metabolism	Alanine, aspartate and glutamate metabolism	ko00250	204
Metabolism	Global and overview maps	2-Oxocarboxylic acid metabolism	ko01210	199
Metabolism	Amino acid metabolism	Phenylalanine metabolism	ko00360	195
Genetic information processing	Translation	Aminoacyl-tRNA biosynthesis	ko00970	183
Metabolism	Carbohydrate metabolism	Pentose phosphate pathway	ko00030	173
Metabolism	Lipid metabolism	Glycerophospholipid metabolism	ko00564	159
Metabolism	Lipid metabolism	Fatty acid degradation	ko00071	146

Environmental information processing	Signal transduction	Phosphatidylinositol signaling system	ko04070	138
Metabolism	Lipid metabolism	Fatty acid biosynthesis	ko00061	137
Metabolism	Lipid metabolism	Biosynthesis of unsaturated fatty acids	ko01040	132
Metabolism	Amino acid metabolism	Phenylalanine, tyrosine and tryptophan biosynthesis	ko00400	132
Metabolism	Carbohydrate metabolism	Inositol phosphate metabolism	ko00562	130
Metabolism	Lipid metabolism	Glycerolipid metabolism	ko00561	129
Metabolism	Carbohydrate metabolism	Pentose and glucuronate interconversions	ko00040	129
Genetic information processing	Replication and repair	Nucleotide excision repair	ko03420	128
Genetic information processing	Folding, Sorting and degradation	Protein export	ko03060	128
Metabolism	Lipid metabolism	alpha-Linolenic acid metabolism	ko00592	127
Metabolism	Replication and repair	DNA replication	ko03030	125
Metabolism	Energy metabolism	Nitrogen metabolism	ko00910	125
Metabolism	Carbohydrate metabolism	Fructose and mannose metabolism	ko00051	123
Metabolism	Amino acid metabolism	Valine, leucine and isoleucine degradation	ko00280	120
Metabolism	Energy metabolism	Photosynthesis - antenna proteins	ko00196	119
Metabolism	Energy metabolism	Sulfur metabolism	ko00920	117
Metabolism	Metabolism of terpenoids and polyketides	Terpenoid backbone biosynthesis	ko00900	117
Metabolism	Carbohydrate metabolism	Galactose metabolism	ko00052	114
Metabolism	Metabolism of cofactors and vitamins	Porphyrin and chlorophyll metabolism	ko00860	112
Metabolism	Glycan biosynthesis and metabolism	N-Glycan biosynthesis	ko00510	93

Metabolism	Carbohydrate metabolism	Propanoate metabolism	ko00640	92
Metabolism	Carbohydrate metabolism	Ascorbate and aldarate metabolism	ko00053	89
Metabolism	Lipid metabolism	Steroid biosynthesis	ko00100	88
Metabolism	Metabolism of other amino acids	beta-Alanine metabolism	ko00410	87
Metabolism	Amino acid metabolism	Tyrosine metabolism	ko00350	87
Metabolism	Replication and repair	Mismatch repair	ko03430	86
Metabolism	Replication and repair	Homologous recombination	ko03440	85
Organismal systems	Environmental adaptation	Circadian rhythm - plant	ko04712	83
Genetic information processing	Transcription	RNA polymerase	ko03020	82
Metabolism	Metabolism of other amino acids	Cyanoamino acid metabolism	ko00460	81
Metabolism	Amino acid metabolism	Valine, leucine and isoleucine biosynthesis	ko00290	81
Metabolism	Metabolism of cofactors and vitamins	Pantothenate and CoA biosynthesis	ko00770	80
Genetic information processing	Transcription	Basal transcription factors	ko03022	79
Genetic information processing	Folding, Sorting and degradation	SNARE interactions in vesicular transport	ko04130	77
Metabolism	Amino acid metabolism	Lysine degradation	ko00310	71
Metabolism	Metabolism of cofactors and vitamins	One carbon pool by folate	ko00670	71
Metabolism	Lipid metabolism	Sphingolipid metabolism	ko00600	71
Cellular processes	Transport and catabolism	Regulation of autophagy	ko04140	69
Genetic information processing	Replication and repair	Base excision repair	ko03410	67
Environmental information processing	Membrane transport	ABC transporters	ko02010	66

Metabolism	Biosynthesis of other secondary metabolites	Flavonoid biosynthesis	ko00941	66
Metabolism	Metabolism of other amino acids	Selenocompound metabolism	ko00450	66
Metabolism	Amino acid metabolism	Tryptophan metabolism	ko00380	65
Metabolism	Amino acid metabolism	Histidine metabolism	ko00340	62
Metabolism	Metabolism of terpenoids and polyketides	Carotenoid biosynthesis	ko00906	58
Metabolism	Metabolism of cofactors and vitamins	Nicotinate and nicotinamide metabolism	ko00760	56
Metabolism	Metabolism of cofactors and vitamins	Ubiquinone and other terpenoid-quinone biosynthesis	ko00130	55
Metabolism	Lipid metabolism	Fatty acid elongation	ko00062	54
Metabolism	Amino acid metabolism	Lysine biosynthesis	ko00300	53
Metabolism	Biosynthesis of other secondary metabolites	Tropane, piperidine and pyridine alkaloid biosynthesis	ko00960	52
Metabolism	Carbohydrate metabolism	Butanoate metabolism	ko00650	50
Metabolism	Lipid metabolism	Arachidonic acid metabolism	ko00590	46
Metabolism	Lipid metabolism	Ether lipid metabolism	ko00565	44
Metabolism	Lipid metabolism	Linoleic acid metabolism	ko00591	43
Metabolism	Biosynthesis of other secondary metabolites	Isoquinoline alkaloid biosynthesis	ko00950	41
Metabolism	Biosynthesis of other secondary metabolites	Stilbenoid, diarylheptanoid and gingerol biosynthesis	ko00945	41
Metabolism	Metabolism of cofactors and vitamins	Biotin metabolism	ko00780	40

Metabolism	Metabolism of cofactors and vitamins	Folate biosynthesis	ko00790	36
Metabolism	Metabolism of cofactors and vitamins	Thiamine metabolism	ko00730	35
Metabolism	Carbohydrate metabolism	C5-Branched dibasic acid metabolism	ko00660	33
Metabolism	Metabolism of cofactors and vitamins	Vitamin B6 metabolism	ko00750	33
Metabolism	Glycan biosynthesis and metabolism	Glycosylphosphatidylinositol(GPI)-anchor biosynthesis	ko00563	26
Metabolism	Glycan biosynthesis and metabolism	Other glycan degradation	ko00511	26
Metabolism	Metabolism of terpenoids and polyketides	Zeatin biosynthesis	ko00908	25
Metabolism	Lipid metabolism	Cutin, suberine and wax biosynthesis	ko00073	23
Genetic information processing	Folding, Sorting and degradation	Sulfur relay system	ko04122	22
Metabolism	Metabolism of cofactors and vitamins	Riboflavin metabolism	ko00740	21
Metabolism	Global and overview maps	Degradation of aromatic compounds	ko01220	20
Metabolism	Metabolism of terpenoids and polyketides	Diterpenoid biosynthesis	ko00904	20
Metabolism	Glycan biosynthesis and metabolism	Glycosaminoglycan degradation	ko00531	19
Metabolism	Metabolism of other amino acids	Taurine and hypotaurine metabolism	ko00430	17
Genetic information processing	Replication and repair	Non-homologous end-joining	ko03450	16
Metabolism	Metabolism of terpenoids and polyketides	Sesquiterpenoid and triterpenoid biosynthesis	ko00909	16

Metabolism	Metabolism of terpenoids and polyketides	Brassinosteroid biosynthesis	ko00905	15
Metabolism	Metabolism of terpenoids and polyketides	Monoterpenoid biosynthesis	ko00902	15
Metabolism	Glycan biosynthesis and metabolism	Other types of O-glycan biosynthesis	ko00514	14
Metabolism	Lipid metabolism	Synthesis and degradation of ketone bodies	ko00072	13
Metabolism	Glycan biosynthesis and metabolism	Glycosphingolipid biosynthesis - globo series	ko00603	12
Metabolism	Metabolism of terpenoids and polyketides	Limonene and pinene degradation	ko00903	10
Metabolism	Biosynthesis of other secondary metabolites	Caffeine metabolism	ko00232	9
Metabolism	Metabolism of cofactors and vitamins	Lipoic acid metabolism	ko00785	8
Metabolism	Biosynthesis of other secondary metabolites	Anthocyanin biosynthesis	ko00942	7
Metabolism	Biosynthesis of other secondary metabolites	Flavone and flavonol biosynthesis	ko00944	7
Metabolism	Glycan biosynthesis and metabolism	Glycosphingolipid biosynthesis - ganglio series	ko00604	5
Metabolism	Biosynthesis of other secondary metabolites	Glucosinolate biosynthesis	ko00966	2

Metabolism	Biosynthesis of other secondary metabolites	Benzoxazinoid biosynthesis	ko00402	1
Metabolism	Biosynthesis of other secondary metabolites	Betalain biosynthesis	ko00965	1
Metabolism	Biosynthesis of other secondary metabolites	Carbapenem biosynthesis	ko00332	1

STable 3 KEGG pathway of the differential expression genes

KEGG Pathway description	pathway ID	Unigene number
Ribosome	ko03010	155
Carbon metabolism	ko01200	98
Protein processing in endoplasmic reticulum	ko04141	88
Biosynthesis of amino acids	ko01230	85
Oxidative phosphorylation	ko00190	84
Photosynthesis	ko00195	61
Spliceosome	ko03040	45
Phagosome	ko04145	43
RNA transport	ko03013	43
Starch and sucrose metabolism	ko00500	42
Glutathione metabolism	ko00480	42
Cysteine and methionine metabolism	ko00270	41
Endocytosis	ko04144	41
Glyoxylate and dicarboxylate metabolism	ko00630	40
Fatty acid metabolism	ko01212	40
Fatty acid metabolism	ko00330	38
Carbon fixation in photosynthetic organisms	ko00710	37
Glycolysis / Gluconeogenesis	ko00010	37
Purine metabolism	ko00230	35

Proteasome	ko03050	35
Pyrimidine metabolism	ko00240	35
Plant hormone signal transduction	ko04075	33
Glycine, serine and threonine metabolism	ko00260	32
Peroxisome	ko04146	31
Pyruvate metabolism	ko00620	30
Ubiquitin mediated proteolysis	ko04120	30
Photosynthesis - antenna proteins	ko00196	30
Amino sugar and nucleotide sugar metabolism	ko00520	27
Biosynthesis of unsaturated fatty acids	ko01040	27
2-Oxocarboxylic acid metabolism	ko01210	24
Alanine, aspartate and glutamate metabolism	ko00250	24
Citrate cycle (TCA cycle)	ko00020	24
Fatty acid degradation	ko00071	23
Phenylpropanoid biosynthesis	ko00940	22
Ascorbate and aldarate metabolism	ko00053	21
Phenylalanine metabolism	ko00360	21
alpha-Linolenic acid metabolism	ko00592	20
Ribosome biogenesis in eukaryotes	ko03008	17
Plant-pathogen interaction	ko04626	17
Protein export	ko03060	17
mRNA surveillance pathway	ko03015	17
Pentose and glucuronate interconversions	ko00040	16
Valine, leucine and isoleucine degradation	ko00280	15
Nitrogen metabolism	ko00910	15

Sulfur metabolism	ko00920	15
Pentose phosphate pathway	ko00030	15
Inositol phosphate metabolism	ko00562	14
Fatty acid biosynthesis	ko00061	14
Aminoacyl-tRNA biosynthesis	ko00970	14
Glycerophospholipid metabolism	ko00564	13
Tryptophan metabolism	ko00380	13
Phosphatidylinositol signaling system	ko04070	13
Fructose and mannose metabolism	ko00051	12
RNA polymerase	ko03020	12
Terpenoid backbone biosynthesis	ko00900	12
RNA degradation	ko03018	12
DNA replication	ko03030	12
beta-Alanine metabolism	ko00410	11
Flavonoid biosynthesis	ko00941	11
Phenylalanine, tyrosine and tryptophan biosynthesis	ko00400	11
Tyrosine metabolism	ko00350	11
Steroid biosynthesis	ko00100	10
Valine, leucine and isoleucine biosynthesis	ko00290	10
One carbon pool by folate	ko00670	10
Cyanoamino acid metabolism	ko00460	10
Nucleotide excision repair	ko03420	9
Histidine metabolism	ko00340	9
SNARE interactions in vesicular transport	ko04130	9
Galactose metabolism	ko00052	8

Lysine degradation	ko00310	8
Nicotinate and nicotinamide metabolism	ko00760	8
Glycerolipid metabolism	ko00561	8
Thiamine metabolism	ko00730	8
Selenocompound metabolism	ko00450	7
Pantothenate and CoA biosynthesis	ko00770	7
ABC transporters	ko02010	7
Circadian rhythm - plant	ko04712	7
Mismatch repair	ko03430	7
Arachidonic acid metabolism	ko00590	7
N-Glycan biosynthesis	ko00510	7
Propanoate metabolism	ko00640	6
Carotenoid biosynthesis	ko00906	6
Vitamin B6 metabolism	ko00750	6
Homologous recombination	ko03440	6
Porphyrin and chlorophyll metabolism	ko00860	6
Isoquinoline alkaloid biosynthesis	ko00950	5
Sphingolipid metabolism	ko00600	5
Ubiquinone and other terpenoid-quinone biosynthesis	ko00130	5
Regulation of autophagy	ko04140	5
C5-Branched dibasic acid metabolism	ko00660	5
Tropane, piperidine and pyridine alkaloid biosynthesis	ko00960	5
Linoleic acid metabolism	ko00591	5
Butanoate metabolism	ko00650	4

Diterpenoid biosynthesis	ko00904	4
Folate biosynthesis	ko00790	4
Fatty acid elongation	ko00062	4
Degradation of aromatic compounds	ko01220	4
Other types of O-glycan biosynthesis	ko00514	3
Ether lipid metabolism	ko00565	3
Zeatin biosynthesis	ko00908	3
Brassinosteroid biosynthesis	ko00905	3
Lysine biosynthesis	ko00300	3
Basal transcription factors	ko03022	3
Riboflavin metabolism	ko00740	2
Glucosinolate biosynthesis	ko00966	2
Limonene and pinene degradation	ko00903	2
Cutin, suberine and wax biosynthesis	ko00073	2
Taurine and hypotaurine metabolism	ko00430	2
Sesquiterpenoid and triterpenoid biosynthesis	ko00909	2
Stilbenoid, diarylheptanoid and gingerol biosynthesis	ko00945	2
Biotin metabolism	ko00780	2
Monoterpenoid biosynthesis	ko00902	1
Caffeine metabolism	ko00232	1
Lipoic acid metabolism	ko00785	1
Base excision repair	ko03410	1
Flavone and flavonol biosynthesis	ko00944	1

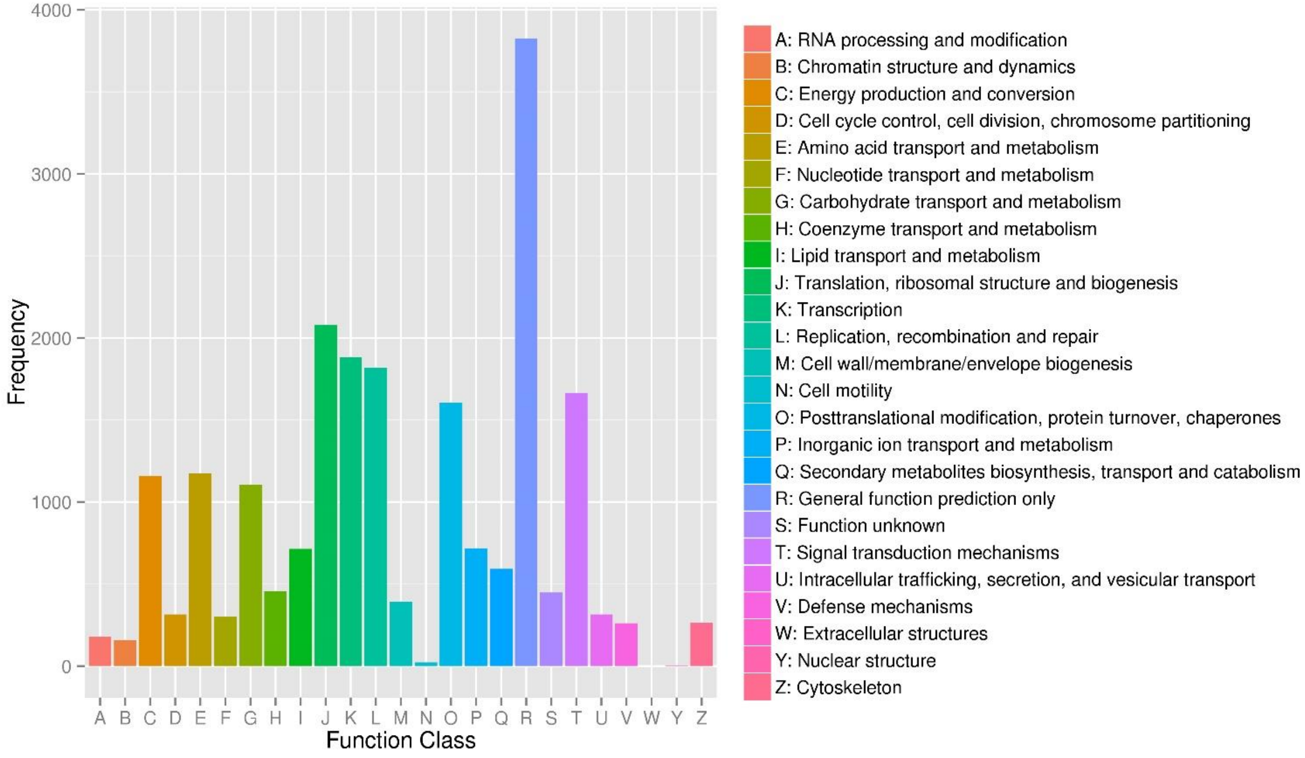

SFigure 1 COG function classification of consensus sequence

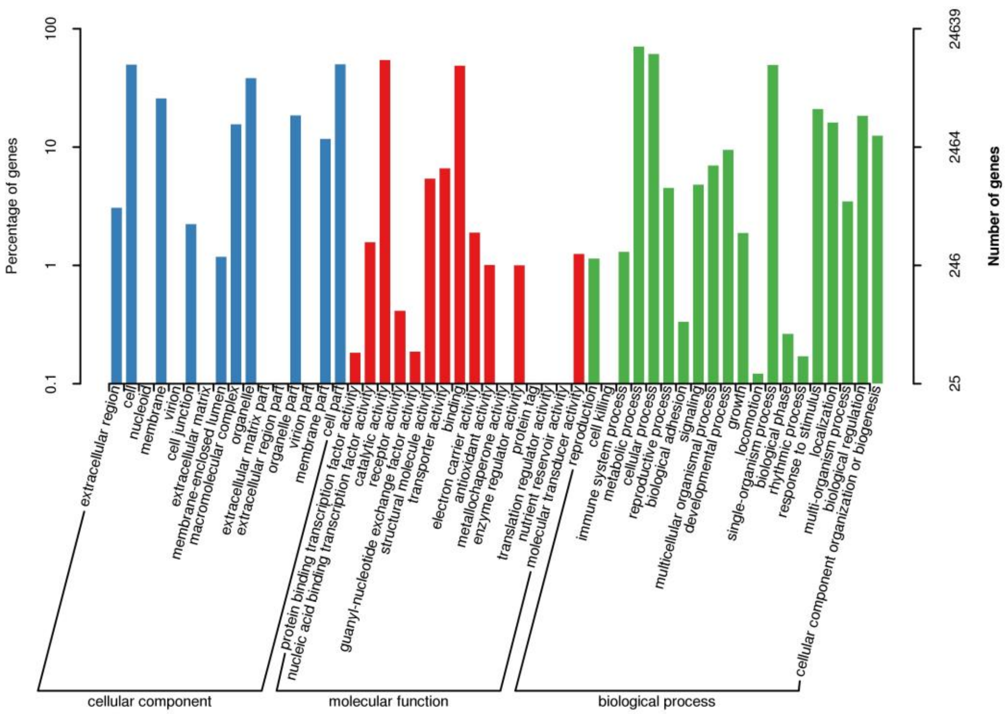

SFigure 2 Gene ontology (GO) classification of assembled transcripts

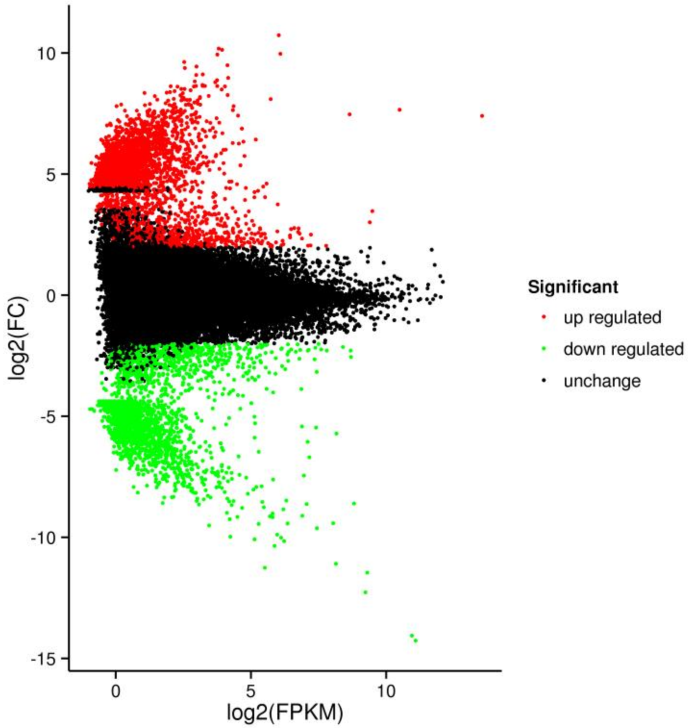

SFigure 3 Volcano plot of differentially expressed genes

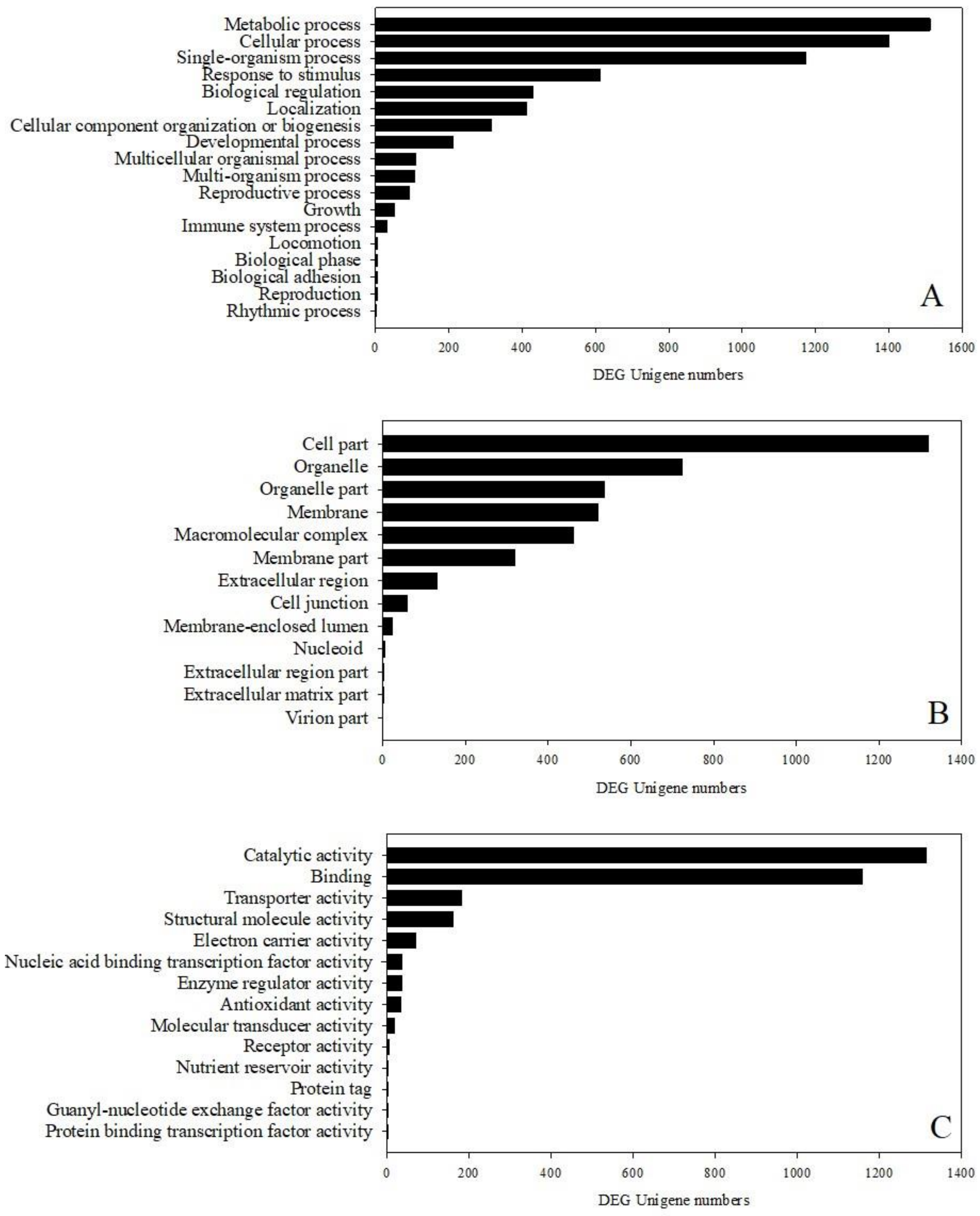

SFigure 4 GO annotation of different expression genes

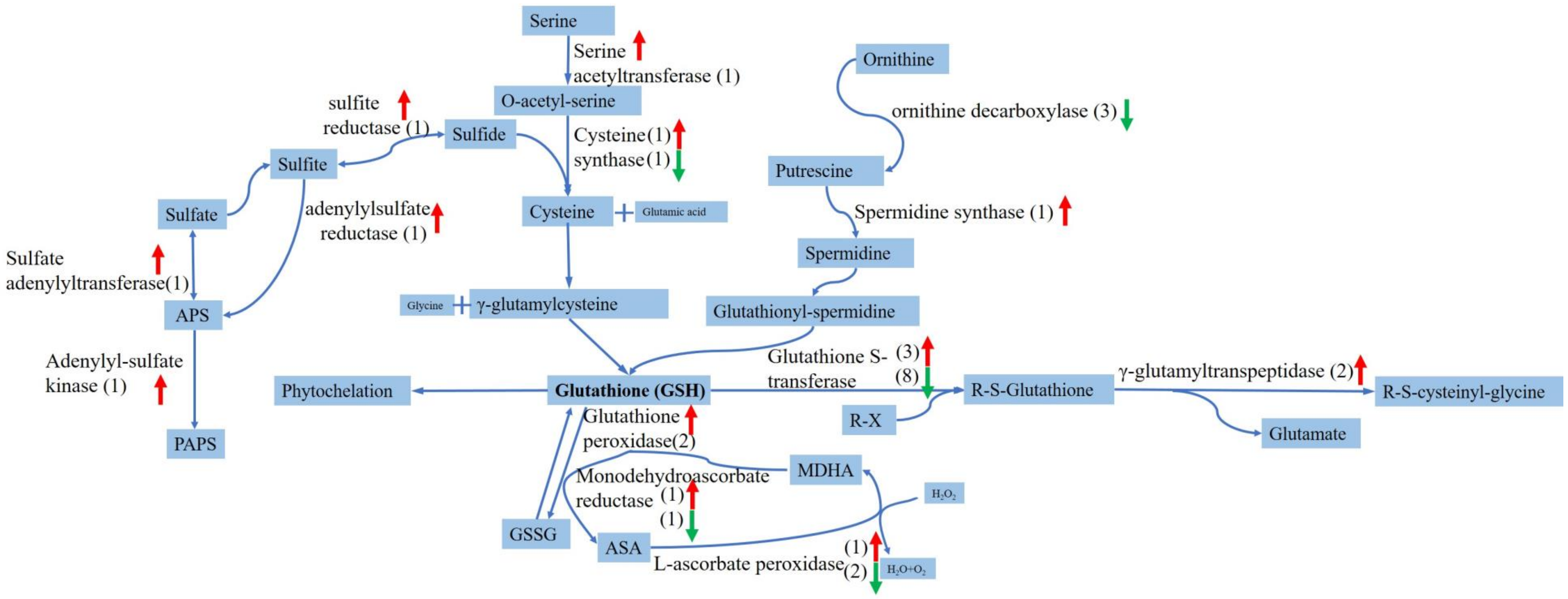

SFigure 5 Transcriptional changes in genes involved in GSH metabolism

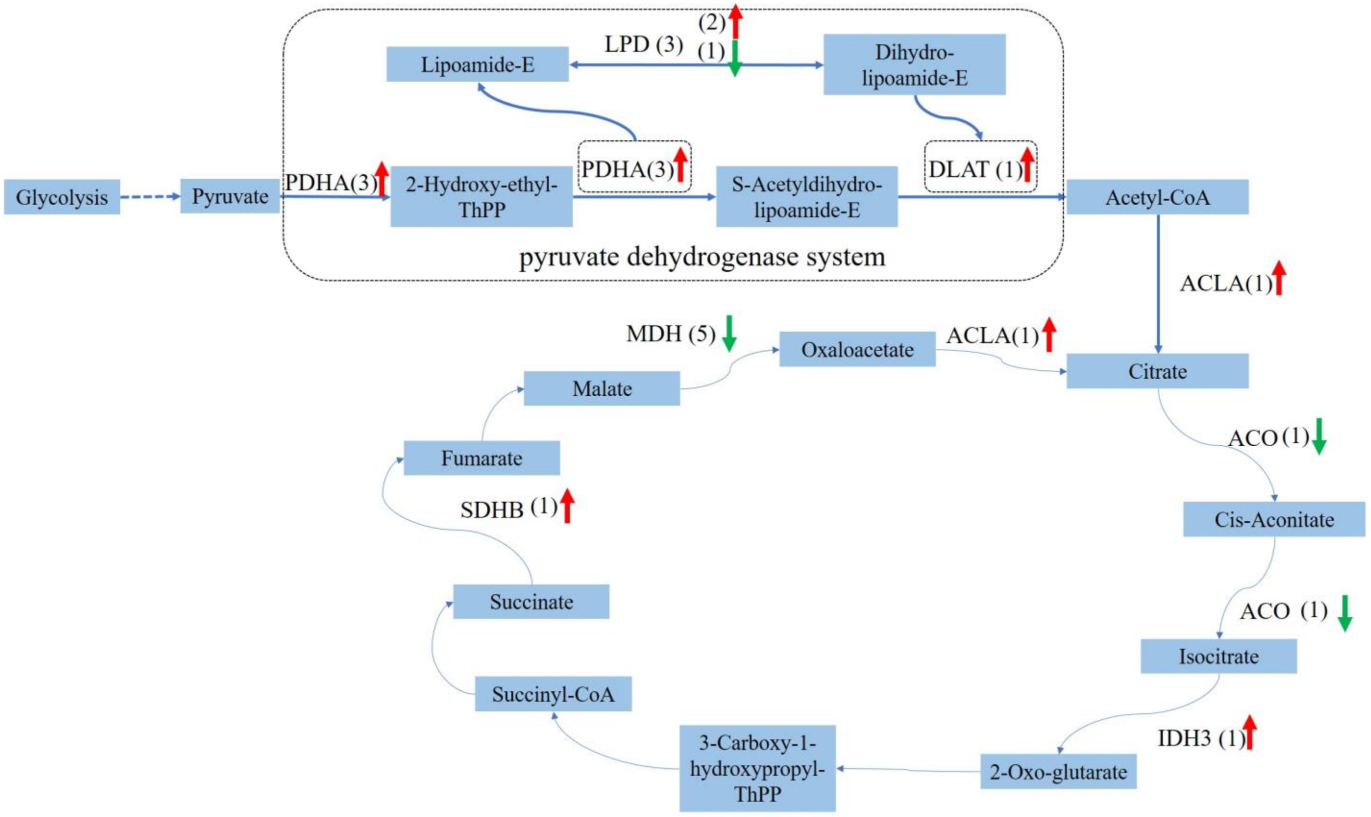

SFigure 6 Transcriptional changes in genes involved in TCA pathway

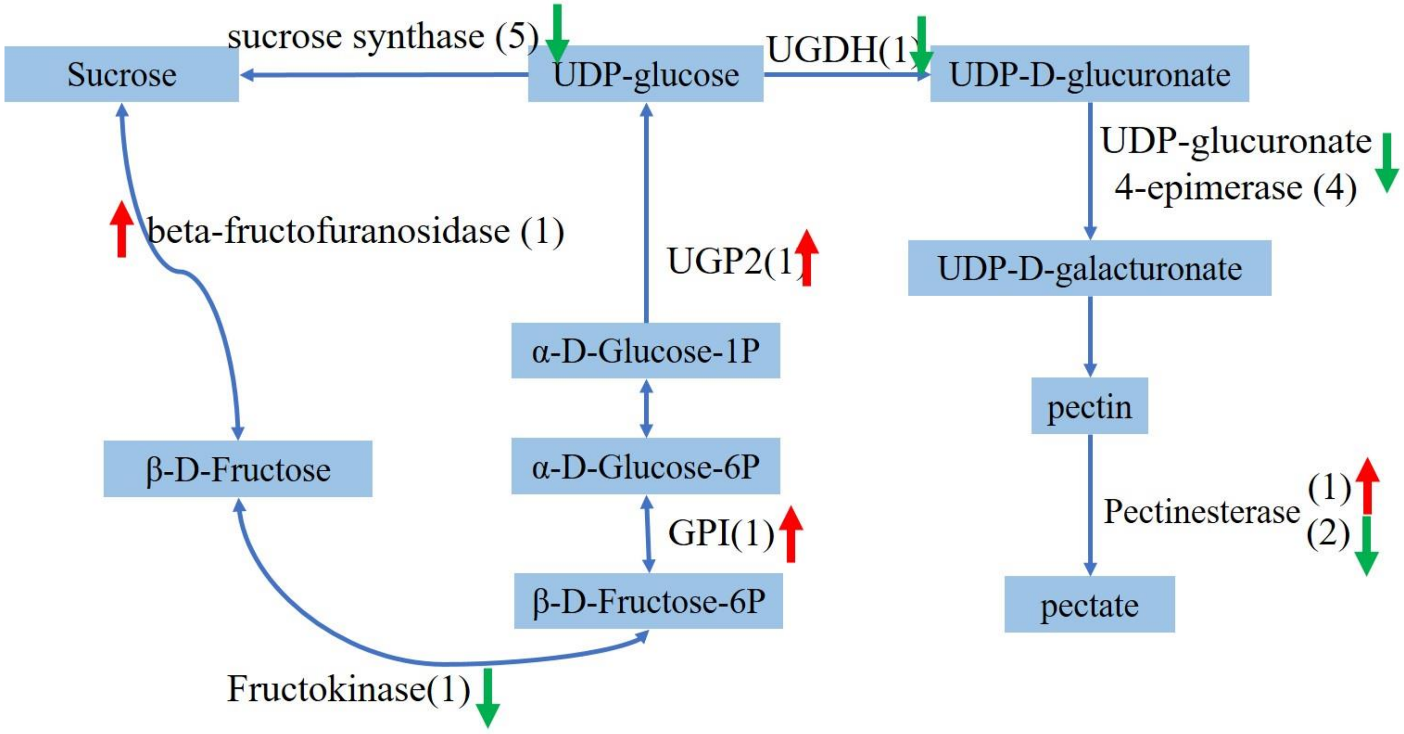

SFigure 7 Transcriptional changes in genes involved in glucose metabolism
